# Correcting for Blood Arrival Time in Global Mean Regression Enhances Functional Connectivity Analysis of Resting State fMRI-BOLD Signals

**DOI:** 10.3389/fnhum.2016.00311

**Published:** 2016-06-28

**Authors:** Sinem B. Erdoğan, Yunjie Tong, Lia M. Hocke, Kimberly P. Lindsey, Blaise deB Frederick

**Affiliations:** ^1^McLean Imaging Center, McLean HospitalBelmont, MA, USA; ^2^Department of Psychiatry, Harvard Medical SchoolBoston, MA, USA; ^3^Department of Radiology, University of CalgaryCalgary, AB, Canada

**Keywords:** resting state networks, BOLD fMRI, functional connectivity analysis, systemic oscillations, global signal regression, systemic noise removal

## Abstract

Resting state functional connectivity analysis is a widely used method for mapping intrinsic functional organization of the brain. Global signal regression (GSR) is commonly employed for removing systemic global variance from resting state BOLD-fMRI data; however, recent studies have demonstrated that GSR may introduce spurious negative correlations within and between functional networks, calling into question the meaning of anticorrelations reported between some networks. In the present study, we propose that global signal from resting state fMRI is composed primarily of systemic low frequency oscillations (sLFOs) that propagate with cerebral blood circulation throughout the brain. We introduce a novel systemic noise removal strategy for resting state fMRI data, “dynamic global signal regression” (dGSR), which applies a voxel-specific optimal time delay to the global signal prior to regression from voxel-wise time series. We test our hypothesis on two functional systems that are suggested to be intrinsically organized into anticorrelated networks: the default mode network (DMN) and task positive network (TPN). We evaluate the efficacy of dGSR and compare its performance with the conventional “static” global regression (sGSR) method in terms of (i) explaining systemic variance in the data and (ii) enhancing specificity and sensitivity of functional connectivity measures. dGSR increases the amount of BOLD signal variance being modeled and removed relative to sGSR while reducing spurious negative correlations introduced in reference regions by sGSR, and attenuating inflated positive connectivity measures. We conclude that incorporating time delay information for sLFOs into global noise removal strategies is of crucial importance for optimal noise removal from resting state functional connectivity maps.

## Introduction

Over the past two decades, interpreting the spatiotemporal patterns and strengths of correlations in low frequency spontaneous hemodynamic fluctuations (~0.01–0.1 Hz) of blood oxygen level dependent (BOLD) signals has become the focus of many functional magnetic resonance imaging (fMRI) studies, especially during resting state (Fox and Raichle, 2007). Several robust and consistent spatial patterns of coherent low frequency hemodynamic signals have been observed across human subjects (Biswal et al., 1995; Greicius et al., 2003, 2004; Fox et al., 2005, 2006; Fransson, 2005; Damoiseaux et al., 2006; Vincent et al., 2006, 2008; Buckner et al., 2008), even in the absence of external stimuli, and have been identified as “resting state networks.” Recently, investigation of alterations in connectivity strengths within and between these functional networks has gained increasing interest for understanding abnormalities in functional brain organization (Seeley et al., 2009; Dosenbach et al., 2010), which has promising applications for early detection of brain connectivity pathologies in neuropsychiatric (Anand et al., 2005, 2009; Garrity et al., 2007; Greicius et al., 2007; Zhou et al., 2007; Uddin et al., 2008; Whitfield-Gabrieli et al., 2009) and neurological disorders (Greicius et al., 2004; Wang et al., 2007; Zhou et al., 2008; Hedden et al., 2009; Sheline et al., 2010).

Resting state functional connectivity magnetic resonance imaging (fcMRI) analysis is based on the assumption that information exchange between functionally related brain regions causes synchronized neuronal activations which, in turn, result in temporally coherent hemodynamic fluctuations unique to each functional network (Beckmann et al., 2005; Damoiseaux et al., 2006; De Luca et al., 2006). However, a major problem with fcMRI analysis arises from the presence of a common global systemic noise effect that is superimposed on top of many of the functional networks (Fox et al., 2009; Murphy et al., 2009; Van Dijk et al., 2010). Although the origin of such neuronally unrelated, systemic physiology-based systemic interference is not clear (Sassaroli et al., 2012), the frequency spectrum of a significant portion of this global effect, namely the systemic low frequency oscillations (sLFOs), overlaps with frequencies of interest involved in detecting neuronally induced spatiotemporal coherence patterns (~0.01–0.1 Hz). Consequently, sLFOs are widely intermixed with spontaneous neuronal oscillations in the resting state BOLD signals, resulting in inflated, or in other words, spurious positive correlations between brain regions and an increase in apparent functional connectivity strengths (Murphy et al., 2013) which in turn, reduces the specificity for detecting brain regions with neuronal activity related coherent hemodynamic fluctuations in fcMRI analyses.

Most existing physiological noise removal methods have focused on cardiac and respiratory fluctuations, and have treated systemic low frequency noise as an epiphenomenon of these processes, or an aliasing artifact (Glover et al., 2000; Lund et al., 2006; Birn et al., 2008a,b; Chang et al., 2009; Chang and Glover, 2009a,b). However; recent studies suggest that sLFOs may arise from a variety of sources including spontaneous variations of arterial blood pressure (Julien, 2006), vasomotion of vessel walls (Gustafsson, 1993; Aalkjaer et al., 2011) and fluctuations in arterial CO_2_ (Wise et al., 2004; Murphy et al., 2011). Hence; they cannot be solely attributed to an aliasing artifact. Previous work from our group has also demonstrated that sLFOs in the brain differ spatially and temporally from low frequency oscillation signal regressors obtained through modeling of cardiac and respiratory signals and their aliasing effects, and should be treated as an independent physiological phenomenon (Tong et al., 2012; Hocke et al., 2016).

One method which does treat the sLFO signal directly is the use of global signal regression (GSR) as a preprocessing step prior to fcMRI analysis. GSR simply involves regressing out the average signal across all voxels within the brain from voxel-wise BOLD data (Desjardins et al., 2001; Greicius et al., 2003; Fox et al., 2005; Fransson, 2005; Tian et al., 2007; Wang et al., 2007; Fair et al., 2008; Uddin et al., 2009). Such a signal averaging procedure emphasizes the common global systemic variance in low frequency BOLD signals while suppressing local spontaneous fluctuations of neuronal origin. Consequently, application of GSR is considered to enhance the observation of spatial patterns of neuronally induced coherent spontaneous hemodynamic fluctuations. However, recent studies have demonstrated that employing GSR as a preprocessing step for noise removal may cause spurious findings of negatively correlated regions in the brain (Fox et al., 2009; Murphy et al., 2009; Van Dijk et al., 2010). This is mainly attributed to the mathematical bias of mandatory negative correlations introduced through the GSR technique, the details of which have been extensively discussed in previous studies (Vincent et al., 2006; Buckner et al., 2008; Fox et al., 2009; Murphy et al., 2009). Briefly, for seed-based resting state fcMRI analysis, regressing the global signal out of each voxel's time series will mathematically force the distribution of voxel-wise correlation values to the time series of a seed region of interest (ROI) to always have a mean correlation value that is less than or equal to zero. Such a shift in the distribution of correlation strengths toward negative values necessarily introduces false or spurious negative correlations where none may exist or it may artificially increase the magnitude of existing true negative correlations. The introduction of artificial negative correlations has a profound impact on assessing the neurobiological validity of, for example, anticorrelations reported between the default mode network (DMN) and the dorsal attention system or task positive network (TPN) which have been thought to reflect an antagonistic relationship between these two functional systems (Fox et al., 2005; Fransson, 2005). Currently; it is still a matter of debate whether the anticorrelations observed between the DMN and TPN are introduced artificially through GSR methodology or have a neurobiological basis (Buckner et al., 2008; Fox et al., 2009; Van Dijk et al., 2010; Murphy et al., 2013).

Previous work from our group has presented compelling evidence that sLFOs, the major constituent of low frequency global systemic noise overlying resting state functional networks (Tong and Frederick, 2010, 2014; Birn, 2012; Murphy et al., 2013), are intrinsic natural signals that travel with blood to all parts of the body (Tong and Frederick, 2012; Tong et al., 2013). sLFOs have been shown to propagate dynamically not only in peripheral tissue with site-dependent temporal delays (Tong et al., 2012); but also throughout the brain with cerebral blood circulation (Tong and Frederick, 2010, 2014; Tong et al., 2011). More specifically, it has been demonstrated that sLFOs obtained from non-brain tissue are widely present in resting state fMRI (rsfMRI) data and they travel with the bulk cerebral blood flow with voxel-specific time delays while the spatiotemporal pattern of their arrival time in each voxel is closely associated with cerebral blood flow circulation patterns (Tong and Frederick, 2010; Tong et al., 2012). sLFOs can also be derived from rsfMRI data with a recursive procedure (Tong and Frederick, 2014), and their flow pattern has been shown to be very consistent with the blood flow patterns obtained via enhanced contrast (DSC) imaging in a recent multimodal, quantitative comparison study (Tong et al., 2016). These studies emphasized the importance of integrating voxel-specific sLFO arrival time information into noise removal strategies for resting state functional connectivity analyses. In previous studies which utilized GSR as a preprocessing step, the low frequency global systemic effect has been modeled as a spatially and temporally homogeneous static phenomenon, and its dynamic passage through the cerebral vasculature has not been taken into account.

In the present study, we test the efficacy of an improved method for removing the global signal from rsfMRI data by taking into consideration a recently well-established physiological observation: dynamic propagation of low frequency systemic oscillations through the cerebral vasculature. We call our method as “**dynamic global signal regression (dGSR)**” and compare its performance in reducing systemic variance from resting state fcMRI maps to the conventional GSR technique, which will be referred to as “**static global signal regression (sGSR)**.”

We propose that global signal calculated from rsfMRI data can be used as a proxy systemic regressor for tracking the dynamic passage of sLFOs through the cerebral vasculature. We hypothesize that applying a voxel-specific optimal time delay to global signal prior to regression will (i) improve modeling and removal of systemic physiological noise and (ii) reduce spurious negative correlations in resting state fcMRI maps. To test our hypothesis, we focus on two systems that are suggested to be intrinsically organized into anticorrelated networks: the DMN and TPN. We evaluate the efficacy of both dGSR and sGSR methods in terms of enhancing specificity and sensitivity of functional connectivity measures, and examine the spatial extent and magnitude of negative and positive correlations with an ROI in posterior cingulate cortex (PCC), a hub region in DMN, when dGSR is applied instead of the conventional sGSR method. Lastly, we discuss some of the potential mechanisms for why the proposed approach provides better performance with supplementary results from simulation data.

It is important to note that by allowing for optimized time delay between the global signal and time series in any voxel, their correlation will by definition be equal to or greater than the correlation when no temporal shifting is applied, which will in turn lead to the removal of more noise variance. However, more importantly, temporally shifting the global signal by an amount which maximizes its cross-correlation with voxel-wise rsfMRI time series prior to regression, may attenuate the introduction of spurious negative or positive correlation measures that results from regressing out a systemic regressor which is aligned with an incorrect time shift with respect to the underlying systemic component of that voxel time series. The reduction in the magnitude of spurious correlations (false and/or inflated positive and negative correlation measures), and the increase in the specificity of neuronal activity related functional connectivity measures are the primary goals of developing this noise removal method.

## Materials and methods

### Subjects and study design

A concurrent resting state near infrared spectroscopy (NIRS) and fMRI study was conducted on 11 healthy volunteers. Two subjects were excluded due to poor signal quality and the results for nine subjects will be shown here for analysis (6 males, 3 females, average age ± SD, 35.6 ± 14). The protocol was approved by the McLean Hospital Institutional Review Board and written informed consents were obtained from all subjects prior to scanning. A 6 min 30 s resting state MRI scan was performed on each subject while NIRS data was simultaneously recorded from a probe placed on the right fingertip. During the resting state scan, subjects were asked to refrain from excessive movements and stay motionless in the scanner while viewing a gray screen with a central fixation point.

### fMRI data acquisition and preprocessing

All MRI data were acquired on a Siemens TIM Trio 3 T scanner (Siemens Medical Systems, Malvern, PA) using a 32-channel phased array head matrix coil. A high-resolution anatomical image set was acquired for slice positioning and coregistration of the functional data (MPRAGE, TR/TI/TE = 2530/1100/3.31, 256 × 256 × 128 voxels over a 256 × 256 × 170 mm sagittal slab, GRAPPA factor of 2). fMRI resting state scans with 32 axial slices were collected with the following parameters: TR/TE = 520/30 ms, flip angle 43°, matrix = 64 × 64, FOV = 220 × 220 mm, multiband factor = 6, 323.5 mm slices with a 0.5 mm gap parallel to the AC–PC line extending down from the top of the brain. For each subject, resting state fMRI data was preprocessed using the “First-level analysis-> Pre-stats” option of FEAT, part of the FMRIB Software Library (FSL; Smith et al., 2004) prior to further analysis. This consisted of discarding the first 20 volumes of each run to allow for T1-equilibration effects, motion correction, slice timing correction, and spatial smoothing with a 5 mm Gaussian kernel. A temporal bandpass filter was applied to retain frequencies in the range of 0.01–0.1 Hz. Spurious or regionally nonspecific variance due to motion was removed by regression of six nuisance variables obtained by rigid body head motion correction. Finally, functional images were registered first to the high resolution anatomical images taken during each scan, and then to a high resolution T1 anatomical template in the Montreal Neurological Institute (MNI) atlas space, MNI152 (Evans et al., 1993) at 2 mm isotropic resolution, by computing and applying affine and nonlinear transforms for each step.

### NIRS data acquisition

NIRS data was collected by means of two MRI compatible NIRS probes, one placed on each subject's forehead over the right prefrontal area and the other placed on the right fingertip. Data from the forehead probe was not used in the present analysis. The fingertip probe had two optical fibers, one source fiber and one detector fiber (with a source-detector distance of 1.5 cm). A near-infrared tissue imager (Imagent, ISS, Inc., Champaign, IL) was placed in the MRI control room and connected to the probe by 10 m optical fibers. The sampling rate of NIRS data acquisition was 6.25 Hz, and two illumination wavelengths (690 and 830 nm) were used. NIRS data was recorded continuously during the MR exam. NIRS signals were converted to time series of concentration changes of oxygenated hemoglobin (HbO) and deoxygenated hemoglobin (HBR) using the Lambert-Beer Law during the post-processing step and band-pass filtered to the low frequency range (0.01–0.1 Hz).

### fMRI data analysis

#### Physiological noise correction methods

For each subject, the average BOLD signal across all voxels in the brain was computed and denoted as “global signal.” Physiological noise in fMRI data was then removed using two regression approaches for the global signal: (1) the conventional **sGSR**, which utilizes the global signal as a regressor for removing systemic effects in a generalized linear model (GLM) analysis; (2) the **dGSR**, which utilizes a voxel-specific optimally delayed version of global signal as a regressor in the GLM. For each voxel, the dynamic global regressor was obtained by shifting the global signal time series with an “optimal” time delay that maximized its cross-correlation with that voxel's BOLD time series. The optimal delay that provides maximum correlation with voxel time series was determined using the “Regressor Interpolation at Progressive Time Delays (RIPTiDe)” procedure with in-house, custom software (Frederick et al., 2012). The time delay analysis software can be downloaded from https://github.com/bbfrederick/rapidtide. The performance of both static and dynamic global regression methods were compared to the case where no physiological correction method was applied to the standard preprocessed fMRI data and this approach was denoted as “no regression.”

#### Resting state fcMRI analysis

fcMRI analysis was performed using a seed based approach (Biswal et al., 1995). After each physiological noise correction method was applied, connectivity maps were obtained by computing correlation between the mean signal time course from voxels within a specific ROI in PCC and the time courses from all acquired voxels using Pearson's correlation coefficient (R). The PCC seed region was determined by drawing a 10 mm sphere centered around a previously published Talairach coordinate (after conversion to MNI coordinates; Shulman et al., 1997; Fox et al., 2005). Correlation coefficients were converted to normally distributed z-scores using Fisher's r-to-z transformation to allow for second level group analysis.

Following Murphy et al.'s procedure (Murphy et al., 2009), the individual subject correlation maps generated after sGSR were thresholded at a correlation value of ± 0.3 (corresponding to *p* < 0.0001 when corrected for multiple comparisons using a Bonferroni correction) to define masks for task negative and task positive regions at the subject level. The mean correlation strengths within the task negative and task positive region masks were computed for both methods and compared to interpret how the strength of negative correlations observed using conventional sGSR are altered when dGSR is applied.

**Figure 1:**
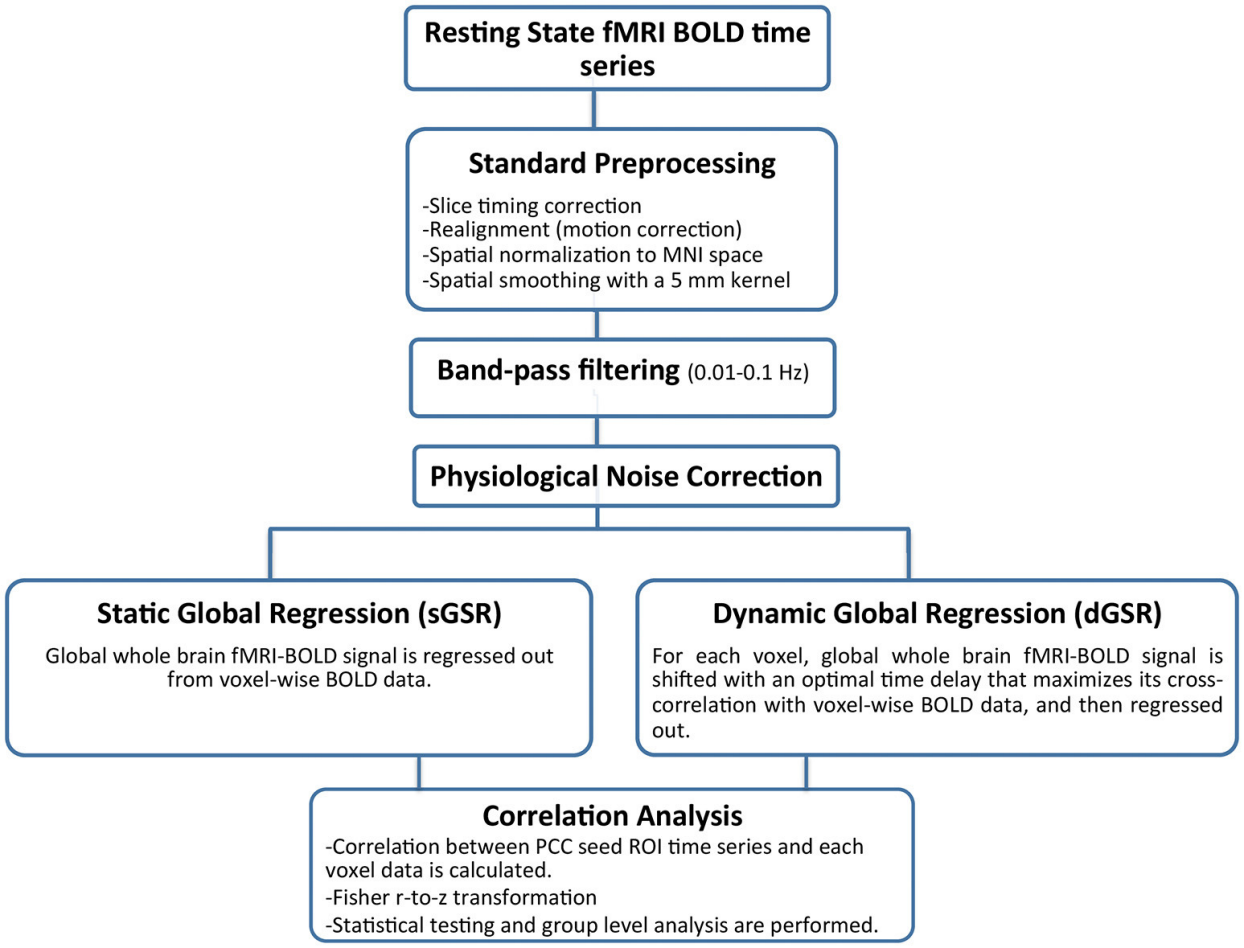
**A schematic representation of the data analysis pipeline**.

For each physiological noise correction method, the group level functional connectivity map was generated by performing a fixed effects one-sample *t*-test across all subjects. The group level z-score maps were corrected for multiple comparisons at a significance level of *p* < 0.05. A schematic representation of the data analysis pipeline is demonstrated in Figure 1.

#### Performance evaluation

We hypothesized that applying a voxel-specific optimal time delay to the global signal prior to regression would improve modeling and removal of systemic physiological noise from resting state fMRI data and reduce spurious negative and positive correlations in fcMRI maps. This hypothesis was tested both on real and simulated data.

To compare the performance of dGSR and the conventional sGSR methods in terms of functional network detection, we focused on two systems that are suggested to be intrinsically organized into anticorrelated networks: the task negative network (TNN), or in other words DMN, which consists of brain regions that exhibit coherent spontaneous fluctuations in the absence of a task and deactivation during cognitive tasks (Shulman et al., 1997; Raichle et al., 2001); and the TPN which has been shown to exhibit coherent hemodynamic fluctuations during cognitive and attentional tasks (Fox et al., 2005; Fransson, 2005). We created ROIs for representative subregions that were positively or negatively correlated with the PCC seed, a major hub region in DMN, following Fox et al. (2005). The TNN ROIs (regions positively correlated with PCC) were created in medial prefrontal cortex (MPFC), and the left and right lateral parietal cortex (LLPC, RLPC). The TPN ROIs included frontal eye field (FEF), left and right insula [Insula (L), Insula (R)], left and right intraparietal sulcus [IPS (L) and IPS (R)], left and right dorsolateral prefrontal cortex [DLPFC (L) and DLPFC (R)], medial temporal lobule (MT) and supplementary motor area (SMA; Chai et al., 2012).

The efficacy of dGSR and sGSR methods in modeling and removing physiological noise was evaluated by comparing three metrics: (i) % variance explained with static and dynamic global regressors, (ii) magnitude (sensitivity), and (iii) specificity of connectivity measures in selected ROIs after each regression method is applied.

##### Explained variance

**Figure 2:**
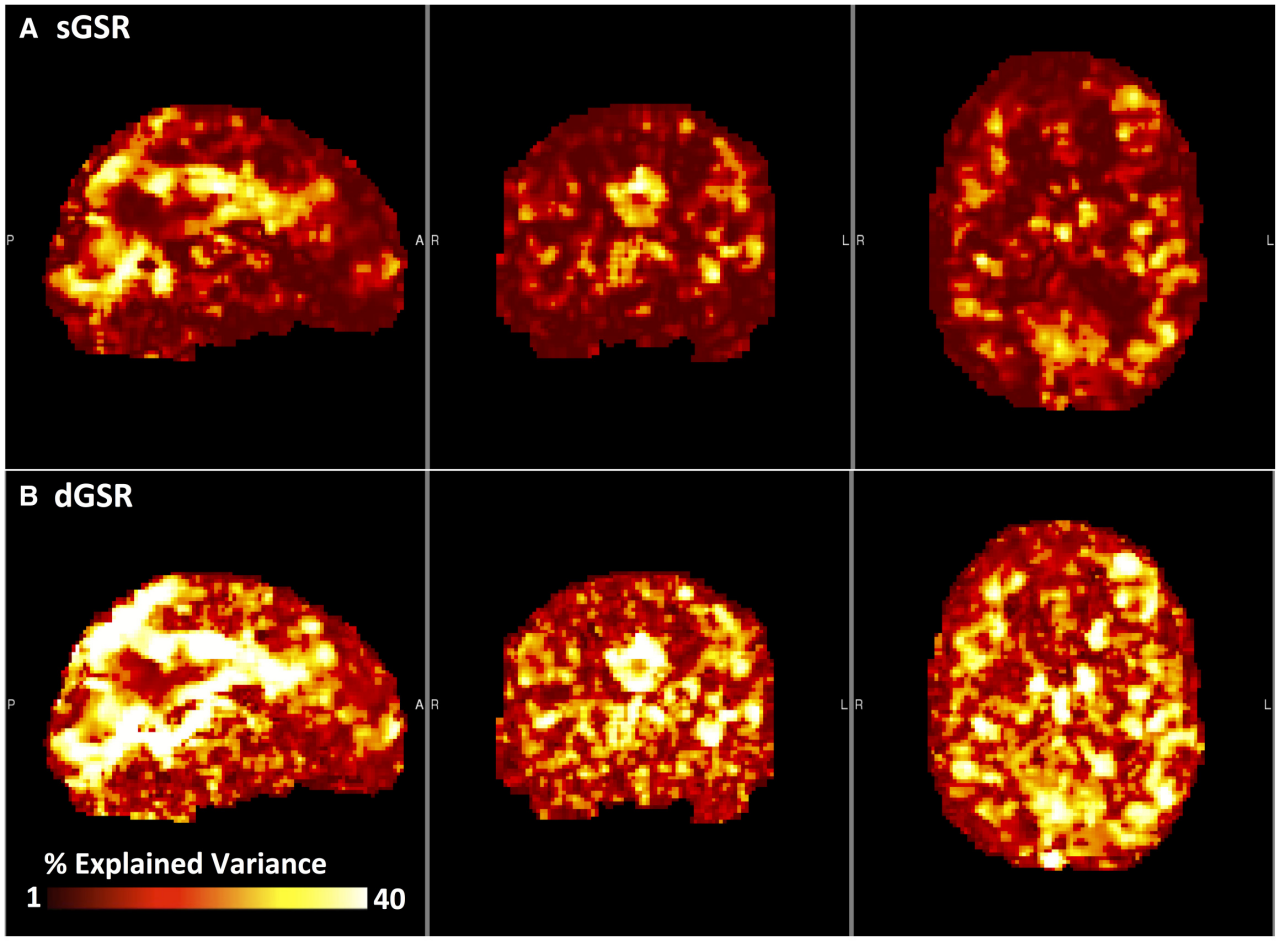
**Percent explained variance map computed by using static (A) and dynamic (B) global regressors is illustrated for a representative subject**.

For each subject's fMRI data; the correlation maps obtained by correlating each voxel time series with static and dynamic global regressors were squared and multiplied by 100 to determine the percentage of signal variance explained in each voxel. For a quantitative comparison, the mean explained variance over all voxels was calculated for both regression methods at the subject level (**Figure 5A**). For each subject, a spatial map of the additional variance explained by dGSR was generated by subtracting the sGSR variance map (Figure 2A) from the variance map obtained with dGSR (Figure 2B). These single subject additional variance maps were averaged to produce a group level map showing the spatial distribution of improvements offered by dGSR (**Figure 5B**).

One potential concern with the dGSR method is whether *any* aperiodic or periodic low frequency signal (such as a sinusoidal wave) could remove equal or similar amounts of variance when an optimal time delay is applied prior to regression. To test this hypothesis, we applied the same dynamic regression procedure by (a) using periodic low frequency sine waves as noise regressors and (b) randomly swapping subjects' global signals with each other and employing dGSR with a different subject's low frequency global signal regressor (**Figure 6**). Here, we should note that applying a band-pass pass filter and an optimal alignment to any two random signals will result in an inflation of correlation measures between them. Therefore; false positive correlations can be calculated with the significance thresholds obtained from traditional statistical methods. This phenomenon has been well investigated in a recent study (Hocke et al., 2016). To prevent the chance of obtaining any spurious significant correlations between optimally aligned systemic model regressors and voxel-wise signals, the lower threshold for significance of the maximum correlation coefficient was calculated with a Monte Carlo simulation procedure and found to be R_*thr*_ = 0.28 at α = 0.01. In other words, a voxel time series will be considered valid for regression only if the initial correlation with low pass filtered and time shifted systemic regressor is above this threshold.

##### Specificity comparison

We examined the specificity of the two preprocessing strategies for detecting correlations and anticorrelations with a metric which describes the absolute correlation strength for each ROI with respect to an uncorrelated reference region ROI (Weissenbacher et al., 2009). More precisely, we compared the specificity of sGSR and dGSR methods by computing connectivity between the PCC seed and the selected ROIs in TNN (DMN) and TPN regions, and comparing them with connectivity between PCC seed and two reference regions with which no correlation is expected. The reference regions were 10 mm spheres that were centered around MNI coordinates (−30, −88, 0) and (30, 88, 0) corresponding to visual cortex. Following Weissenbacher et al. (2009), we defined specificity as:

(1) $$S_{target}=\frac{\left|Z_{target}\right|-\left|Z_{reference}\right|}{\left|Z_{target}\right|+\left|Z_{reference}\right|}$$

*Z*_*target*_ is the group level Z score from the PCC to the selected ROI, and *Z*_*reference*_ represents the average Z score from PCC to the left and right visual reference regions (with these regions, no significant correlation is expected). Specificity of each target ROI ranges from −1 to 1.

#### Time delay simulations

To demonstrate how sGSR can introduce spurious positive and negative correlation measures in seed based fcMRI analyses, we adopted a time delay simulation scheme very similar to the phase simulations presented by Murphy et al. (2009). In these simulations (**Figure 9**), null time series lasting 1000 time points were generated on a 64 by 64 grid. Keeping in mind that global systemic fluctuations carry a mixture of multiple frequencies in the low frequency range, the resting state systemic fluctuations were modeled with a regressor obtained by bandpass filtering a random Gaussian noise signal (mean zero, standard deviation of 1, length 1000) to the frequency range of 0.01–0.1 Hz. This systemic effect modeling regressor was added to each time series with temporal shifts ranging from 0 to 10 s along the x-axis in equal incremental steps. A time delay range of 10 s was chosen to ensure that we completely cover the physiological time range for blood to complete its cycle from carotid arteries to the jugular veins which takes about 6–9 s (Crandell et al., 1973). Random Gaussian noise with a mean of zero was added to each voxel time series to represent process noise. The standard deviation of this noise varied progressively along the y-axis from 0 to 5 in equal increments. A “neuronal” hemodynamic signal was generated by convolving the canonical hemodynamic response function with a 1000 point stimulus regressor consisting of zeros and ones, with ones representing stimulus presentation. This “neuronal” signal was added to various voxels forming vertical columns (C1–C7) on the 64 by 64 grid to simulate a coherent network representing functionally related regions. The contribution of these neuronally induced signals to the voxel signals in which they reside varied from 0.5 to 4% depending on the noise level. The resulting data set consisted of a 64 by 64 grid of time series with systemic fluctuations of varying temporal shifts in x-axis and increasingly large noise components in y-axis (we avoid to use the term “phase” in this simulation since the systemic fluctuations are aperiodic and carry multiple frequencies, and hence phase would have no meaning). The regions where coherent neuronal signals of different amplitudes were added consisted a total of 14% of the voxels. Global signal was obtained by averaging signals from the 4096 time courses and was tested to be orthogonal to the “neuronal” hemodynamic signal. A 3 by 3 voxel ROI was drawn in voxels where the neuronal signals reside. The average time series in seed ROI was determined prior to regression, and after applying sGSR and dGSR methods. The correlation of each voxel to the seed ROI time series was calculated to generate connectivity maps before and after each method was applied.

These simulations were repeated by keeping all procedures the same; however choosing single frequency periodic sinusoidal waves spanning the 0.01 to 0.1 Hz range whose phase varied progressively in the x-axis from 0 to π/4 (as in the original work of Murphy et al., 2009) to represent systemic physiological effects. We observed the improvement in extracting the true correlations and preventing false negative and positive correlation measures with the dGSR method in a consistent manner and report one example simulation in the Supplementary Material.

## Results

**Figure 3:**
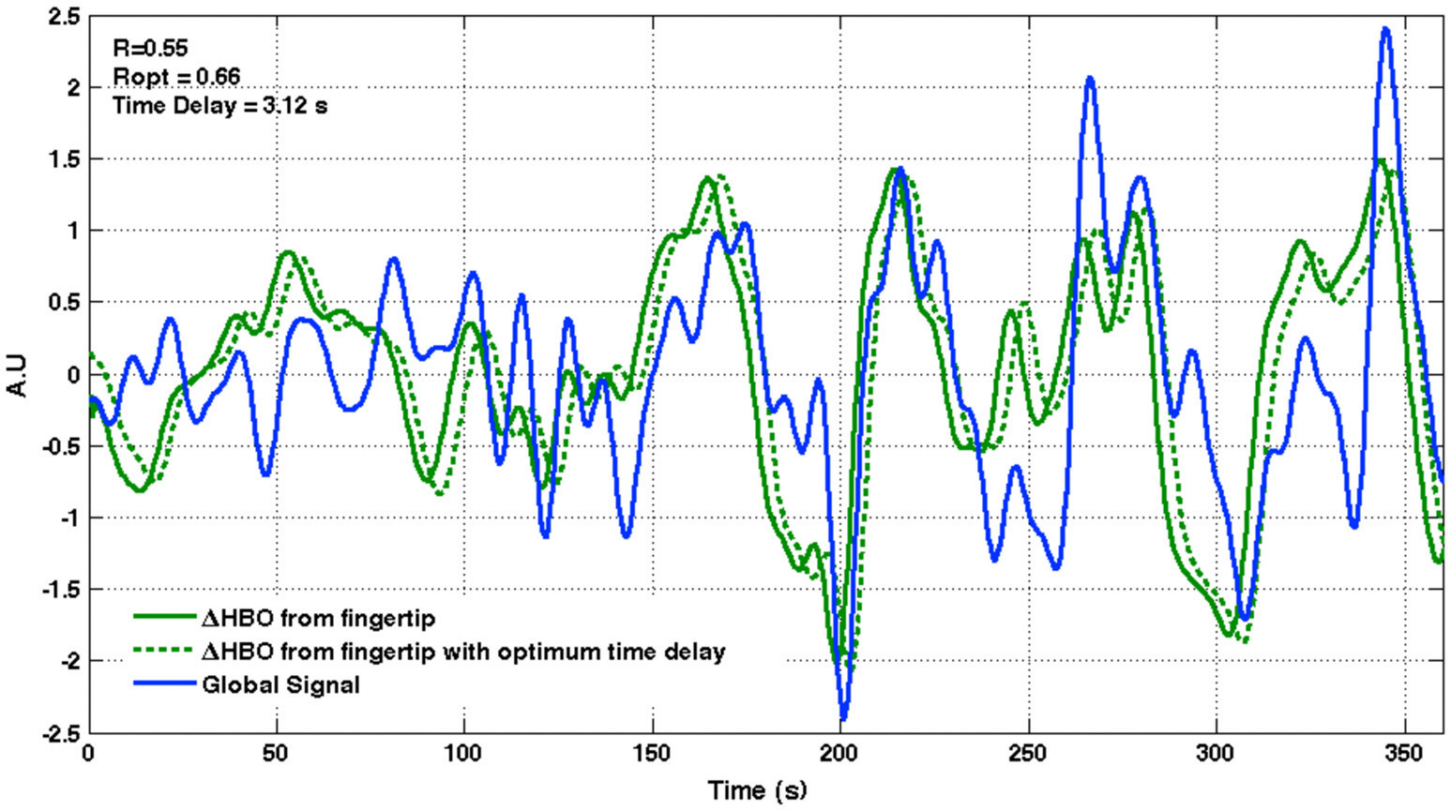
**Low frequency systemic oscillations measured from the periphery with NIRS show a close resemblance to the global signal from fMRI**. Time courses of the low frequency NIRS-HbO signal measured from the fingertip (green) and the whole brain global signal from fMRI-BOLD data are shown for a representative subject, together with an optimally delayed version of the fingertip NIRS-HbO signal (dashed green line). R, correlation between fingertip NIRS-HbO and brain global signal; R_opt_, Maximum cross correlation between the two signals; Time delay, Optimum time delay applied to fingertip NIRS-HbO signal to achieve maximum cross correlation.

**Figure 4:**
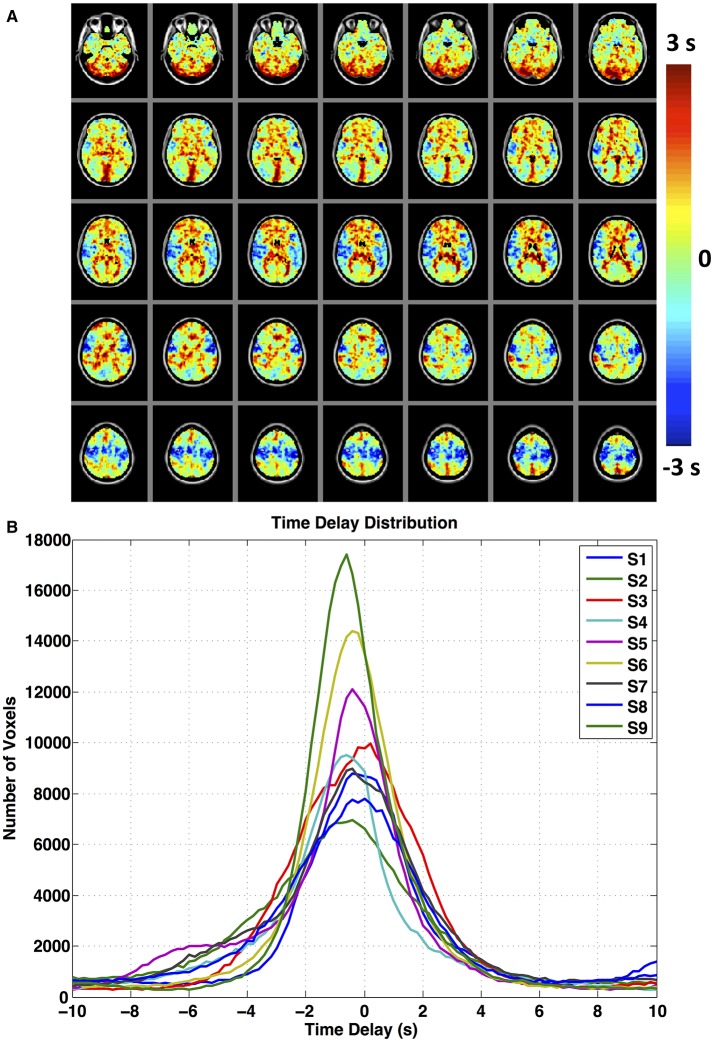
**(A)** Maximum correlation time or time delay map indicates the relative arrival time of global signal to each voxel. Maps represent the group average of the voxel-specific delays in arrival time of the global signal. The delay value that produces the optimal correlation between global signal and voxel-specific BOLD signals varies across space. Both the spatial pattern **(A)** and quantitative distribution **(B)** of the time delay parameter show considerable similarity among subjects.

We begin by showing evidence that the global signal from resting state fMRI is a good representation of the low frequency systemic physiological fluctuations by illustrating its similarity to a peripheral fingertip oxygenation signal of purely non-neuronal origin. Figure 3 presents time courses of the oxygenated hemoglobin (HbO) concentration change measured from the fingertip with a NIRS probe (green) and the whole brain resting state global BOLD signal measured with fMRI (blue) for a representative subject, together with an optimally delayed version of the fingertip signal (dashed green line) so that its correlation with the global signal is maximized. Both systemic signals are band-pass filtered to the low frequency range (0.01–0.1 Hz). Low frequency oscillations (LFOs) measured simultaneously from the fingertip with NIRS reflect purely systemic hemodynamic fluctations with no contribution from neuronal activity and show a good correspondance to the low frequency global signal from resting state fMRI data (*R* = 0.55, *p* < 0.001). Notice how an optimally delayed version of this sLFO signal measured using NIRS, a different modality and in fingertip, a non-brain tissue, closely matches the global signal from fMRI (*R* = 0.65, *p* < 0.001). This observation suggests that systemic LFOs are a major constituent of the global brain fMRI signal in the low frequency range. Table 1 demonstrates correlation at zero time delay (Pearson's correlation coefficient, R), correlation at optimum time delay (maximum cross-correlation denoted as R_opt_) and the time delay that maximizes cross correlation between fingertip NIRS-HbO and the whole brain global fMRI BOLD signal for each subject. All cross-correlation values deemed significant at *p* < 0.01. Improved correlation between global signal from the brain and the sLFO signal measured from the periphery after applying an optimal time delay is consistent with the idea that sLFOs propagate throughout the body, including the brain, with the blood with varying time delays, which has been extensively discussed in our previous work (Tong and Frederick, 2010, 2012; Tong et al., 2013, 2016). Note that the heterogenous lag times likely reflect individual circulatory path differences in the arm and in the carotids, however the range is smaller than the ranges of time delays seen throughout the brain itself (Figure 4).

**Table 1:** **A summary of correlation (R), maximum cross-correlation (R_**opt**_), and time delay measures between the low frequency fingertip NIRS-HbO signal and the whole brain global signal for each subject**.

**Subjects**	***R***	**Ropt**	**Time delay (s)**
S1	0.22	0.39	3.1
S2	0.33	0.6	−5.2
S3	0.55	0.66	3.12
S4	0.33	0.47	−2.6
S5	0.7	0.73	0.4
S6	0.36	0.4	−1.52
S7	−0.23	0.54	3.09
S8	0.16	0.22	3.12
S9	0.57	0.63	1.04
Mean	0.34	0.51	0.75
STD	0.28	0.16	4.9

*The group mean and standard deviation (STD) are shown for each parameter*.

Figure 4A represents the mean spatial map of voxel-specific optimal time delays applied to the global signal, averaged over all subjects. A few points are worth consideration in this figure. The optimal time delay computed for each voxel resulted in a spatial map whose patterns closely matched the dynamic passage of cerebral blood flow through the brain with respect to both flow path and circulation time. Early delays (light green, blue and light blue) could be observed in symmetrical central regions in the top middle section such as the motor cortices which are fed mostly by middle cerebral arteries, while voxels with late time delays were concentrated in the white matter and draining veins. The drainage systems were mostly colored with red and yellow, indicating they are located toward the end of the blood passage. Hence; we believe the time delay parameter obtained with global signal represents the relative arrival time of sLFOs in each voxel. Moreover; the dynamic map shows a good consistency to the cerebral blood flow patterns obtained with DSC bolus track imaging (Tong et al., 2016) and the patterns obtained with recursively generated sLFOs from fMRI (Tong and Frederick, 2014) and from peripheral NIRS recordings (Tong et al., 2012) which is indicative of the physiological relevance and robustness of the procedure. Figure 4B shows a histogram of time delay values for all subjects. The time delay distribution histograms show a remarkable consistency among subjects in the form of normal distributions slightly centered to the left. The full width half maximum (FWHM) values for these relative blood arrival time distributions fall approximately between 4 and 7 s. This range is in compliance with the time ranges measured for cerebral blood to complete a full cycle between the internal carotid artery and the internal jugular vein in healthy subjects in previous studies (Crandell et al., 1973; Schreiber et al., 2002).

### Performance evaluation

#### Explained variance

**Figure 5:**
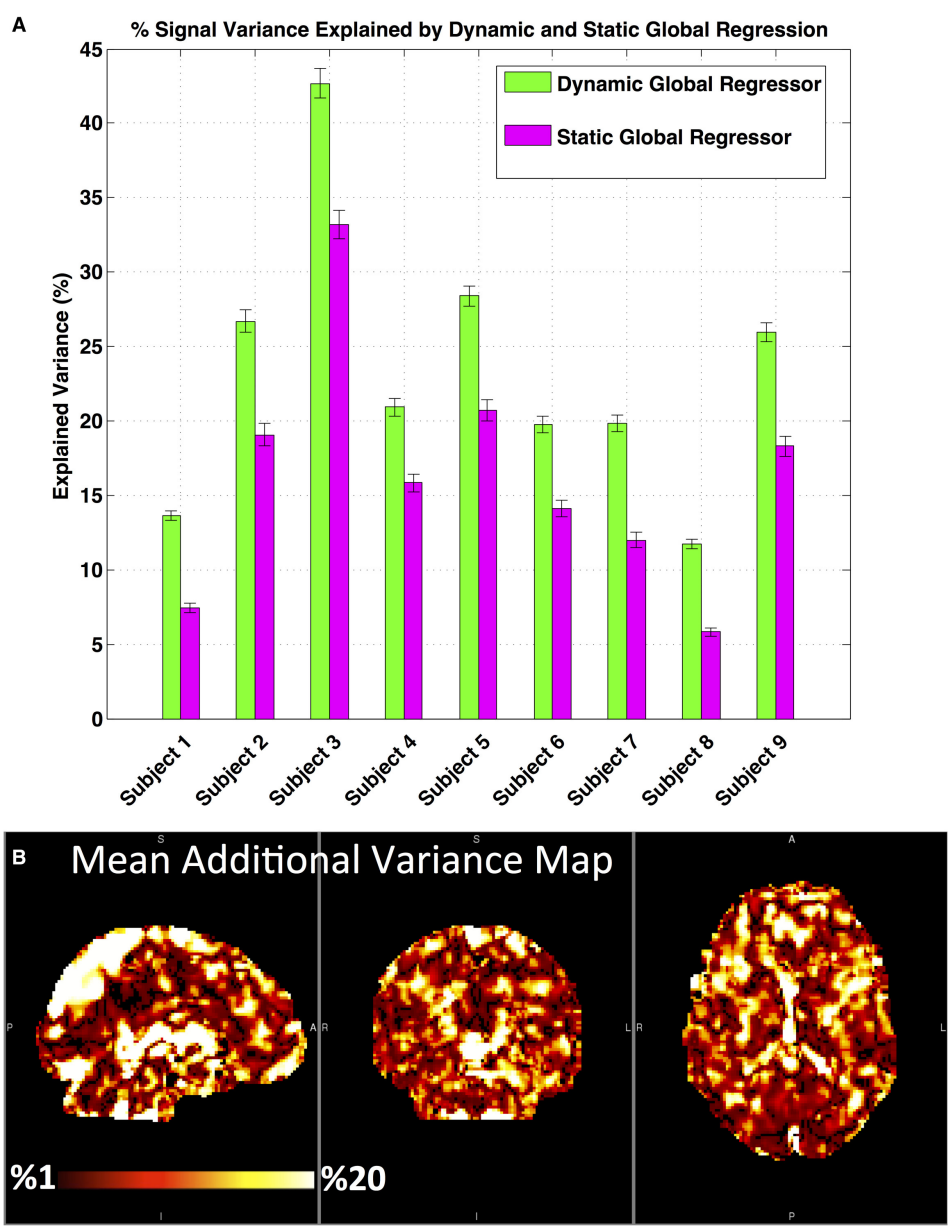
**Percentage signal variance explained by sGSR and dGSR methods**. dGSR explains variance in the data to a greater extent when compared to sGSR. **(A)** Mean % signal variance explained by dynamic and static global regressors. Error bars represent standard error of the mean. **(B)** A spatial map of the mean additional variance explained by voxel-wise optimally delayed (dynamic) global regressor and the static global regressor, averaged over all subjects.

Figure 5 compares the performance of dGSR and sGSR methods in terms of explaining variance in the data. In Figure 5A, an average of the percentage of signal variance explained by static global signal and the optimally delayed dynamic global signal over all brain voxels is demonstrated for each subject. The dynamic global signal explains variance in the data to a greater extent in all subjects (two tailed paired *t*-test, *p* < 0.05), with a mean additional explained variance of 12.5% when compared to the static global signal. Static global signal accounts for a large fraction of the signal variance throughout the brain—up to 30% in some subjects. However, adjusting for blood arrival time differences in each voxel with the dynamic global signal increases the amount of signal variance modeled and removed throughout the brain for all subjects, with particularly large increases in heavily perfused regions in the cortex and in large blood vessels such as the superior sagittal sinus and lateral sinus. Subtracting the static global regression variance map from the dynamic global regression map yields a spatial map of the additional variance explained over the brain volume, shown in Figure 5B. Applying a voxel-specific optimal time delay to the global signal prior to regression leads to an increase in the amount of variance explained in all brain voxels, with particularly large increases in heavily perfused regions in the cortex and in large blood vessels near superior saggital sinus and lateral sinus. Up to 20% additional variance is explained using dGSR in highly perfused regions and near large blood vessels.

**Figure 6:**
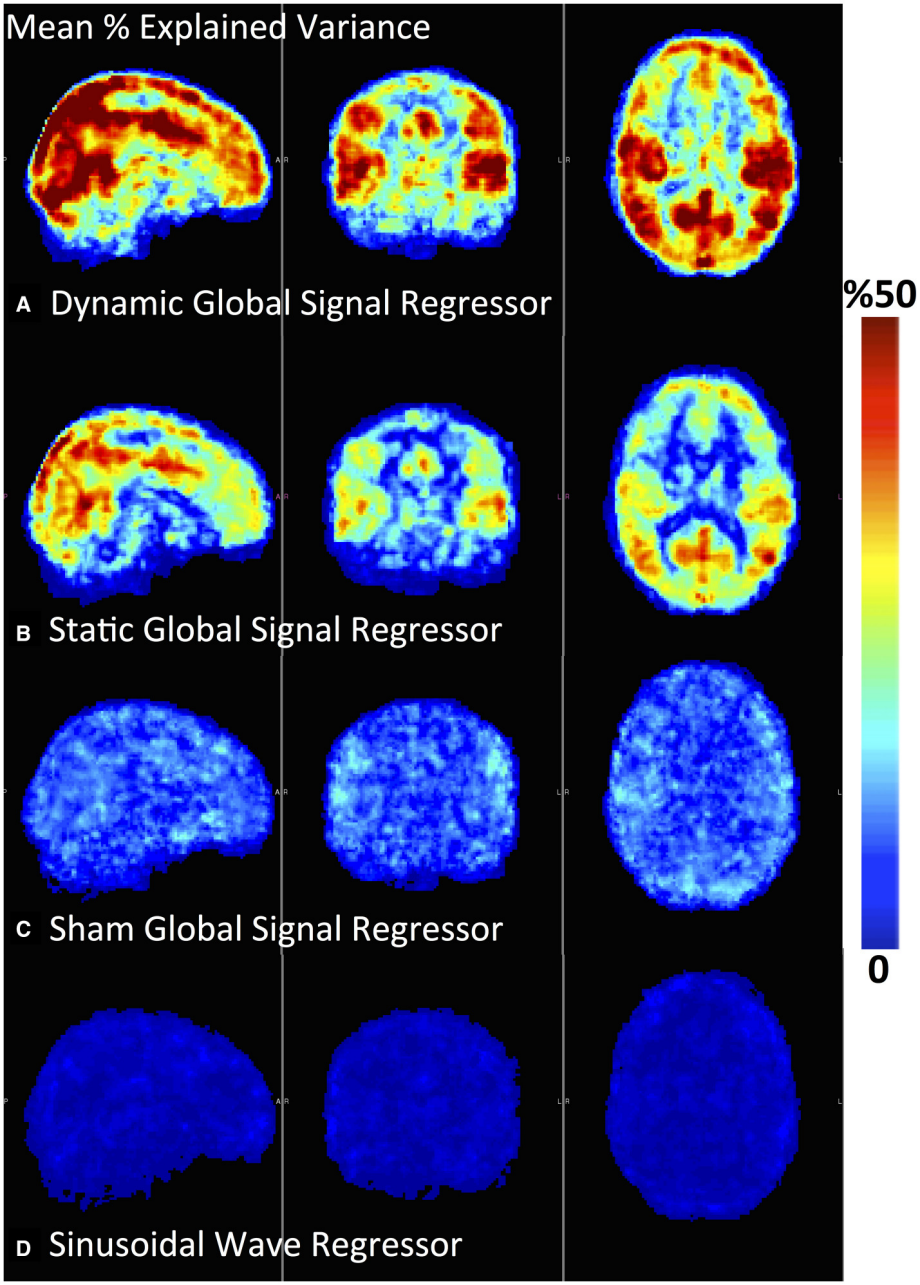
**A spatial map of % Explained Variance averaged across all subjects is shown for (A) Dynamic global signal regressor, (B) Static global signal regressor, (C) Sham global signal regressor (For each subject's resting state fMRI data, explained variance is calculated using another subject's global signal), (D) a Sinusoidal Wave Regressor with a frequency of 0.1 Hz**.

One concern with the dynamic regression approach is whether the same variance removal performance can be obtained by optimally delaying any randomly selected aperiodic (i.e., low frequency global signal of another subject) or periodic low frequency signal (such as a sinusoidal wave). Figure 6 demonstrates a spatial comparison of explained variance performance averaged over all subjects obtained by using (a) a dynamic global regressor, (b) a static global regressor, (c) an optimally delayed sham global regressor, and (d) an optimally delayed low frequency (0.1 Hz) sinusoidal wave regressor. For this data set, 81% of the voxels presented significant correlations with the dynamic global regressor with an overall mean explained variance of 29%. Meanwhile; most of the cross-correlations obtained between voxel time series and swapped (sham) global regressors or periodic sinusoid wave regressors did not pass the statistical significance threshold. Moreover, for the surviving voxels which had significant correlation with the optimally aligned sham global or sine wave regressors, the mean time delay maps did not produce any spatial coherence (Supplementary Material). These results suggest that the spatially consistent time delay maps resembling cerebral circulation and the consequent improvement in systemic variance removal can be achieved only by systemic regressors that are intrinsic to each subject's physiology during data collection.

#### Magnitude of connectivity strengths (sensitivity)

**Figure 7:**
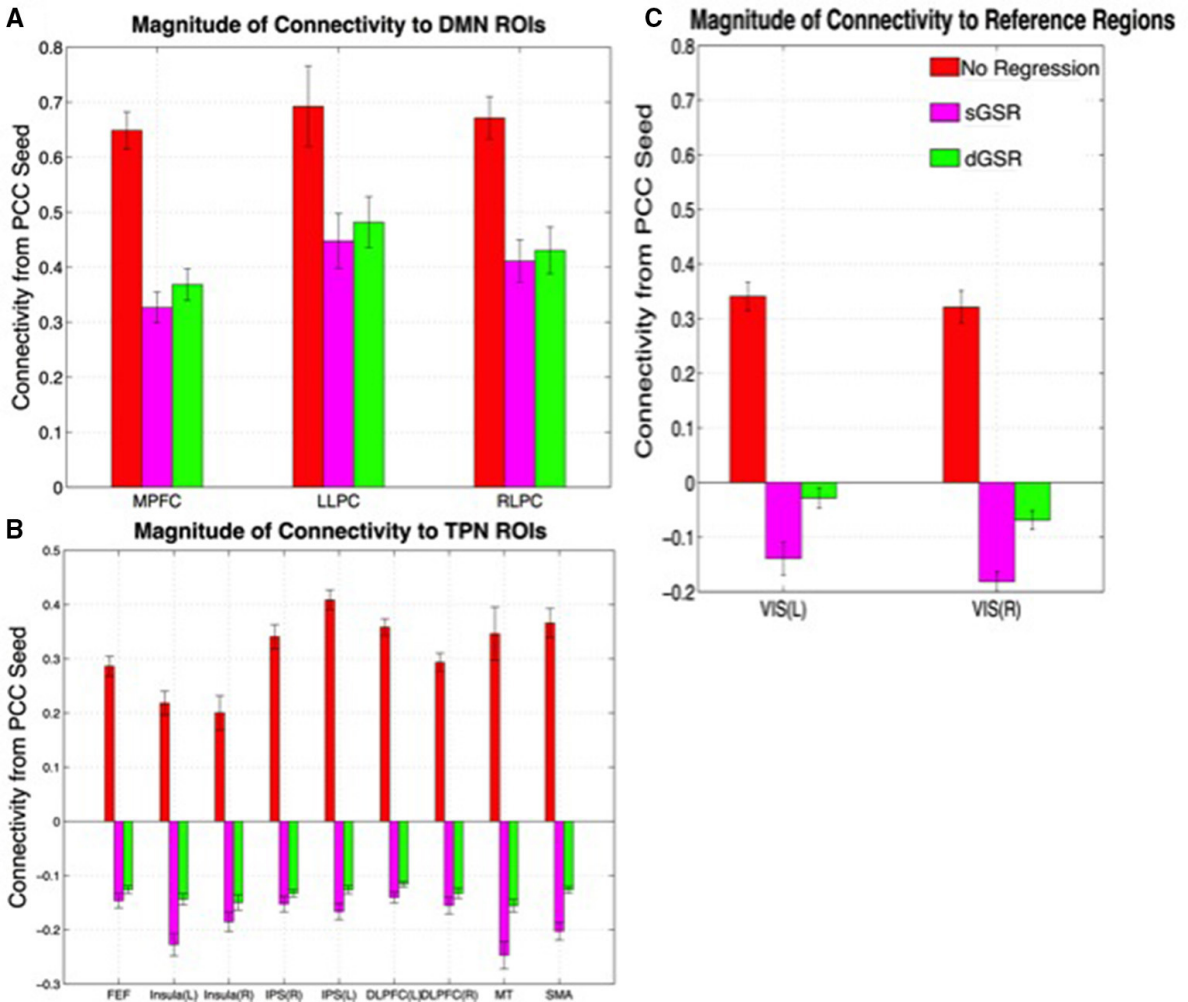
**Group level connectivity strengths from the PCC seed to ROIs in (A) major DMN ROIs (task negative regions), (B) TPN ROIs, (C) reference regions**. L, left hemisphere; R, right hemisphere; VIS, visual cortex ROI.

Figure 7 demonstrates connectivity strengths from the PCC seed to selected ROIs in (i) TNN regions, or in other words major DMN hubs with which significant positive correlations are expected (Figure 7A), (ii) major TPN regions in the dorsal attention system with which anti-correlations have been reported in previous studies (Figure 7B; Chang and Glover, 2009b; Chai et al., 2012) and (iii) reference regions with which no significant correlations are expected (Figure 7C). The connectivity strengths with related ROIs are illustrated after fMRI data is preprocessed with dGSR and sGSR methods and compared to the case when no physiological noise correction is performed.

As shown in Figure 7A, for TNN regions associated with DMN, dGSR results in higher positive correlations between PCC and all of the selected ROIs (*p* < 0.001) when compared to sGSR. After sGSR, the average correlation strength in *z*-values for MPFC, LLPC and RLPC were 0.33 ± 0.03, 0.45 ± 0.05, 0.41 ± 0.04, while after dGSR, the average correlation values for the same ROIs were 0.37 ± 0.03, 0.48 ± 0.05, 0.43 ± 0.04 respectively. When no physiological noise correction was applied, positive correlations were much stronger which suggests that common physiological noise contributes to the overestimation of correlations between these brain regions unless it is removed.

As shown in Figure 7B, for TPN regions, anticorrelations that are computed after sGSR are still observed after dGSR but with much smaller magnitude (Figure 7B). After static global regression, the average correlation strength in *z*-values for FEF, insula (L), insula (R), IPS (L), IPS (R), DLPFC (L), DLPFC (R), MT and SMA ROIs were −0.15 ± 0.01, −0.22 ± 0.02, −0.18 ± 0.02, −0.15 ± 0.02, −0.15 ± 0.01, −0.17 ± 0.01, −0.14 ± 0.01, −0.15 ± 0.02, −0.25 ± 0.02, and −0.2 ± 0.02 while after dynamic global regression, the average correlation values for the same ROIs were −0.12 ± 0.07, −0.14 ± 0.01, −0.15 ± 0.01, −0.413 ± 0.07, −0.13 ± 0.07, −0.12 ± 0.08, −0.13 ± 0.05, −0.15 ± 0.01 and 0.13 ± 001 respectively. When none of the GSR methods are applied, all of the selected ROIs in TPN and DMN are positively correlated with PCC, which is highly indicative of a false positive or type 1 error.

Connectivity from PCC to functionally unrelated regions (in which neither positive nor negative correlations were expected) was assessed using the average connectivity measures between PCC and reference region ROIs (Figure 7C). Artifactual anticorrelation between PCC and reference regions appeared when sGSR was applied (*p* < 0.001, one tailed *t*-test). When no physiological correction method is applied, correlation of PCC with reference regions were inflated, yielding false positives. With the dGSR strategy, neither negative nor positive significant correlations existed with the reference regions. This result is indicative of the efficacy of dGSR method for (a) removing spurious positive correlations due to common systemic noise overlying the two networks and (b) avoiding the spurious negative correlations introduced by sGSR.

#### Specificity

**Figure 8:**
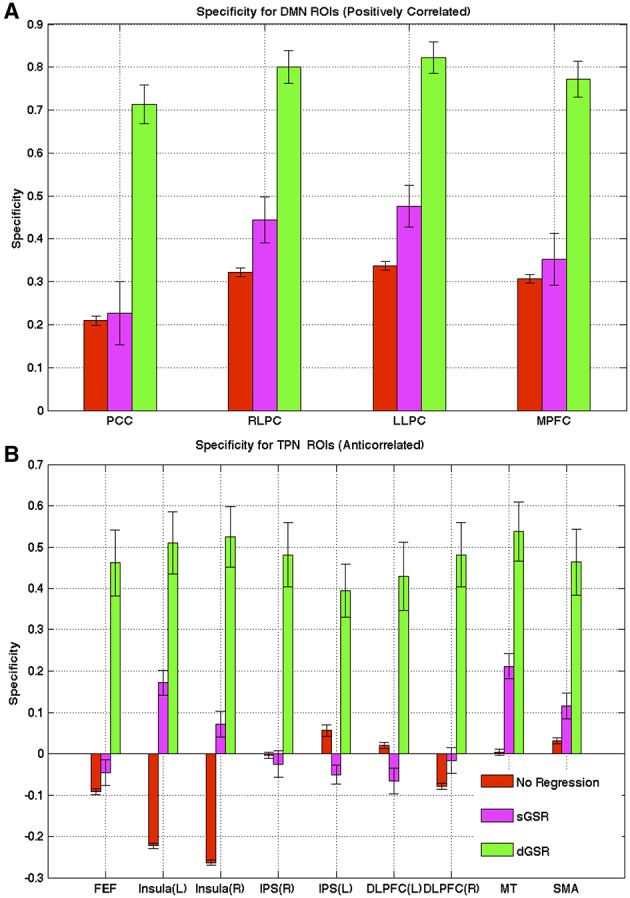
**Specificity of each method for (A) task negative (DMN) and (B) task positive region ROIs**. dGSR method increases connection specificity profoundly in all of the selected ROIs in task positive and task negative network regions when compared to sGSR method (2 tailed paired *t*-test, *p* < 0.05).

Specificity for positively correlated regions with PCC (Figure 8A) was significantly higher (*p* < 0.05, two tailed paired *t*-test) when dGSR was applied instead of sGSR. This finding was consistent for all three DMN hub regions. sGSR did not result in a significant improvement in specificity measures when compared to the case where no physiological correction was applied. For ROIs within the TPN (negatively correlated regions), dGSR resulted in a clear increase in detecting network specific anticorrelations when compared to sGSR (Figure 8B).

#### Time delay simulations

**Figure 9:**
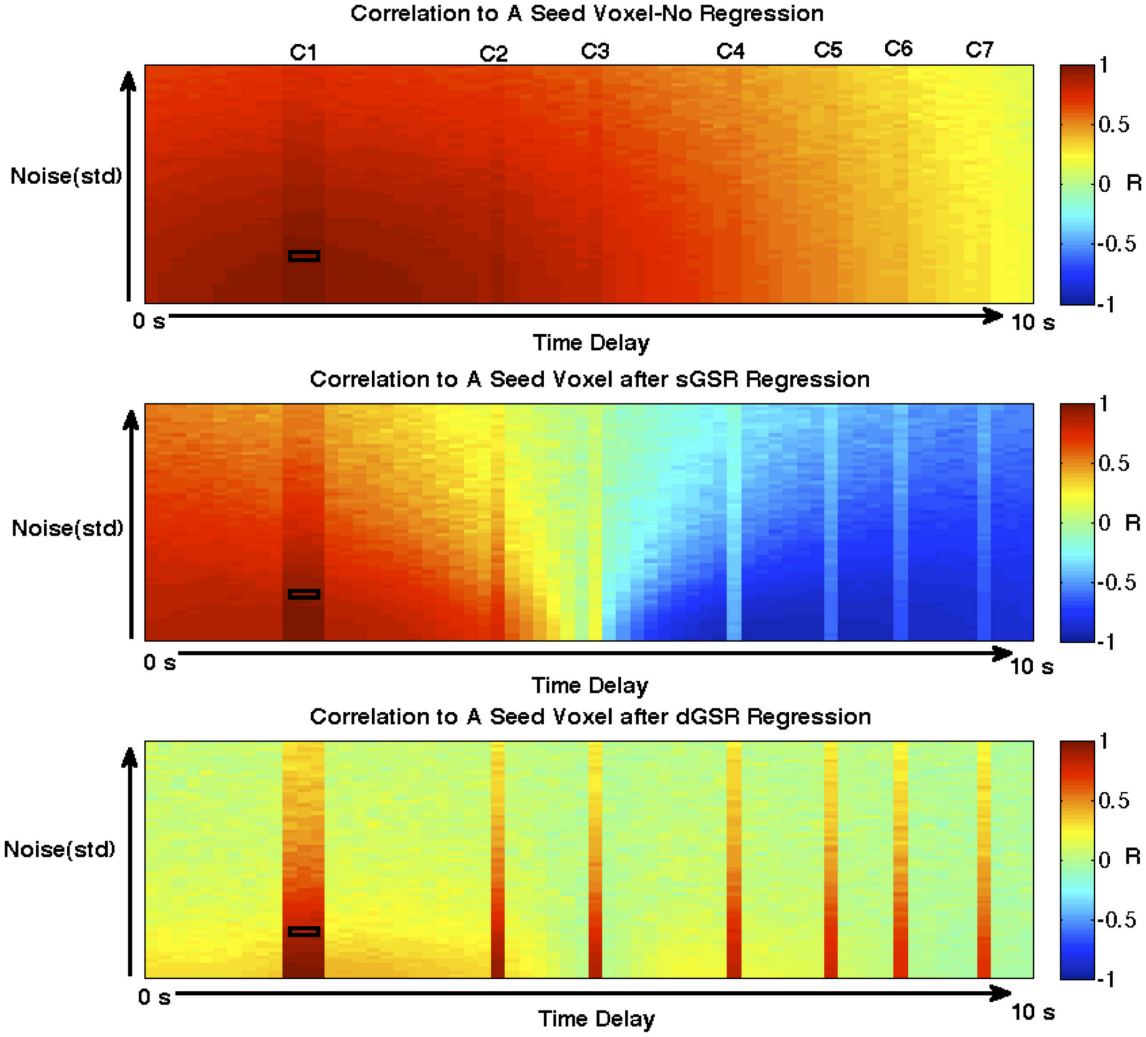
**The results of time delay simulations are illustrated**. The time series for each voxel are generated on a 64 by 64 grid. In each voxel; the systemic component of resting state fluctuations was represented with a band-pass filtered (0.01–0.1 Hz) aperiodic signal which was shifted with time delays ranging from 0 to 10 s along the x-axis. Random Gaussian noise with a mean of 0 and a standard deviation varying from 0 to 5 along the y-axis was added to represent varying levels of process noise. A “neuronal” hemodynamic signal was generated by convolving the canonical hemodynamic response function with a stimulus regressor and was implemented in voxels beneath the columns denoted as C1-C7 to represent a coherent functional network. Each panel displays correlation values to the seed functional ROI (black square) **(A)** when no global signal regression is applied, **(B)** after sGSR, and **(C)** after dGSR. Without global noise regression **(A)** all voxels are correlated on the basis of common systemic global signal. **(B)** When sGSR is performed, approximately half of the voxels become negatively correlated. sGSR may obscure true neuronal correlations by either reducing positive correlation strengths (C2) and making some of these voxels uncorrelated (C3 and C4), or introducing anticorrelations in some regions (including C5, C6, C7) where none should exist. When the dGSR method is applied; all of the reference voxels which are expected to have no significant negative or positive correlations with the seed ROI were uncorrelated after regression. These results are indicative of the improved efficacy of dGSR to attenuate both false positive and false negative correlation measures.

Figure 9 demonstrates time delay simulation results for the three methods. It can easily be observed that when no regression method is applied, the majority of “reference” voxels which have no coherent spontaneous neuronal oscillation content (voxels outside the C1–C7 regions) presented significant positive correlations with the seed ROI time series due to common physiological noise (Figure 9A). When the sGSR method is applied, several points should be noted: (1) Approximately half of the voxels (51%) presented negative correlation with the seed ROI. This finding is in accordance with previous reports by other groups (Fox et al., 2009; Murphy et al., 2009; Weissenbacher et al., 2009) which discussed the mathematical bias of sGSR forcing approximately half of the correlation values to be negative regardless of the initial correlation distribution. (2) About 99% of the voxels which reside outside the depicted network and presented no significant positive or negative correlation with the seed ROI prior to regression (IR_seed, vox_I < 0.28), became significantly negatively correlated after sGSR. This result using simulated data shows that sGSR inflates negative correlation measures and can introduce spurious negative correlations in regions where no correlation is expected. Moreover; some of the voxels which contained coherent spontaneous neuronal fluctuations with the seed ROI became either uncorrelated (C3 and C4 regions) or anticorrelated (C5, C6, C7). This result demonstrates that sGSR may obscure true neuronal network correlations by either reducing positive correlation strength and making some of these voxels uncorrelated, or introducing anticorrelations in some regions where none should exist. sGSR may also underestimate the true positive correlation measures in some regions (C2 region) where coherent neuronal fluctuations do exist. Some of the positive correlation measures were attenuated in the C2 region while some regions containing no neuronal content remained positively correlated with seed ROI (false positives) due to suboptimal noise removal.

When the dGSR method is applied; all of the reference voxels which are expected to have no significant negative or positive correlations with the seed ROI were uncorrelated after regression. This result is indicative of the ability of dGSR to attenuate both false positive and false negative correlation measures. The true correlations with voxels which contained coherent neuronal oscillations were preserved to a good extent as well. These findings using simulated data demonstrate evidence for the improved efficacy of the dGSR approach in (i) preventing inflated spurious negative and positive correlation measures, (ii) preserving true positive correlations, and (ii) eliminating artifactual anticorrelations.

#### Magnitude and spatial extent of positive and negative correlations

**Figure 10:**
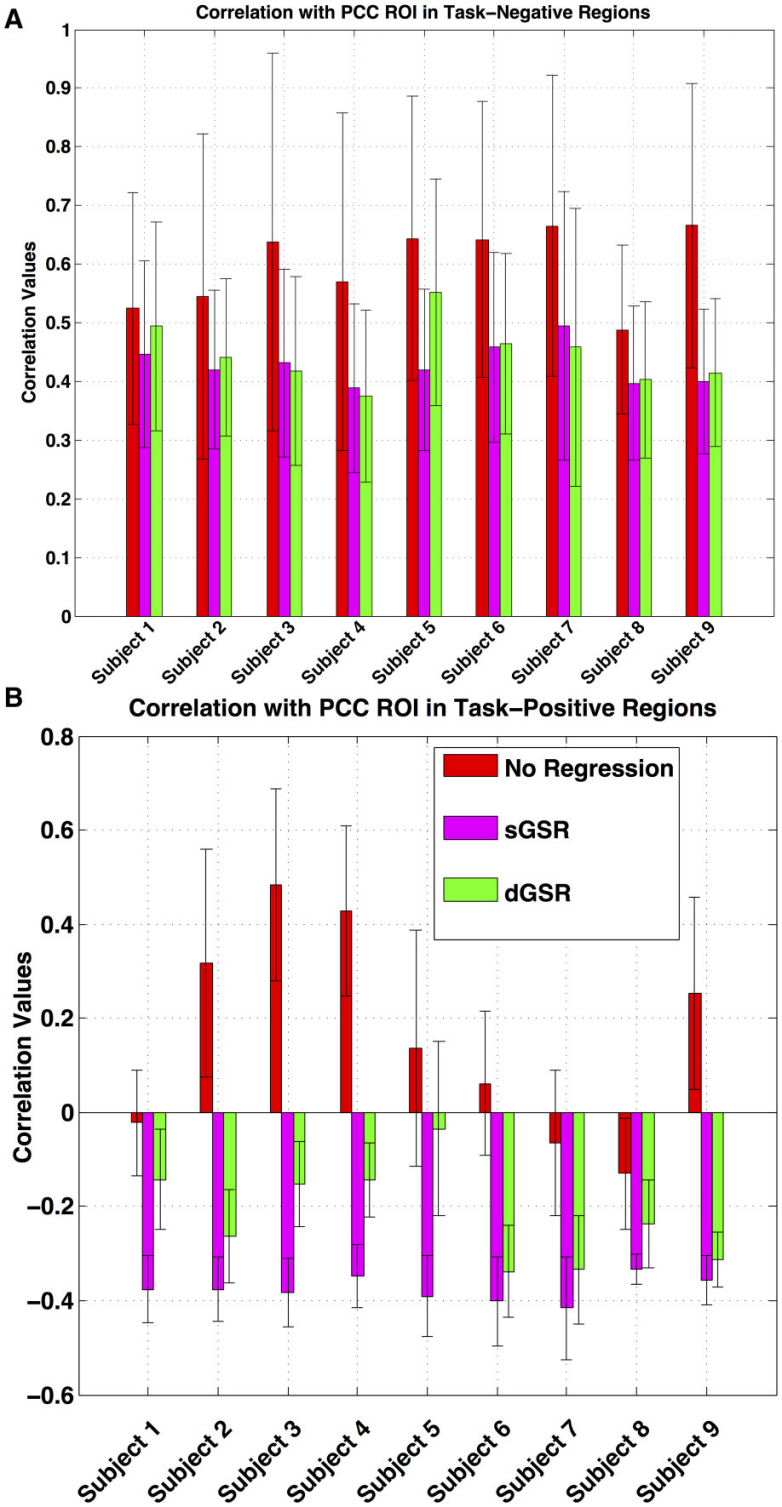
**Average correlation values across task-negative (A) and task-positive regions (B) are shown for all subjects**. Three conditions are depicted: (1) without any physiological correction (red), (2) after sGSR (magenta), and (3) after dGSR (green). Before sGSR, task–positive areas can be highly correlated with the PCC region with only three subjects showing slightly negative correlations. sGSR causes all task-positive areas to become negatively correlated and reduces variability across subjects. When dGSR is applied, an anti-correlated (task-positive) network is still observed but with weaker correlation values.

**Figure 11:**
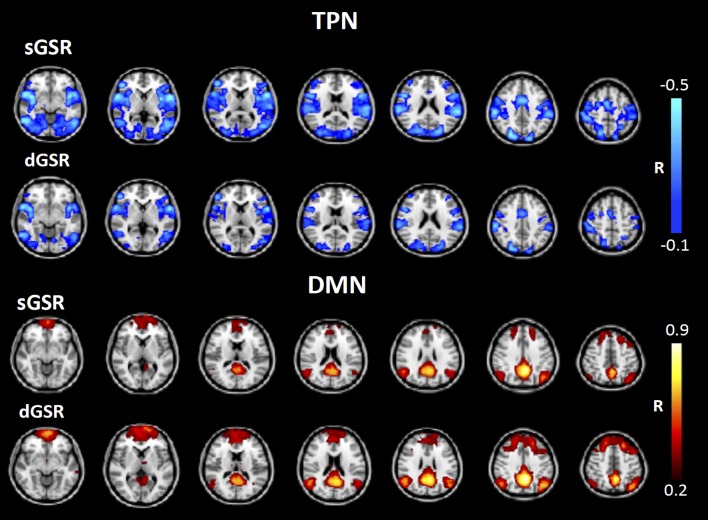
**Functional connectivity measures from PCC to TPN and TNN regions across all subjects (***N*** = 9) after sGSR and dGSR**. sGSR increases the spatial extent and magnitude of negative correlations while reducing the spatial extent and magnitude of positive correlations at the group level.

Figure 10 demonstrates the average correlation values with PCC in TNN and TPN region masks for each subject. For TNN regions (positive correlations with PCC), dGSR and sGSR reduce connectivity strengths to a similar extent when compared to no regression method (no statistically significant difference between the two methods is observed for any of the subjects). However; for TPN regions (negative correlations with PCC, Figure 10B), the mean correlation values with PCC were significantly less in magnitude after dGSR when compared to the mean correlation values obtained after sGSR for each subject (two tailed paired *t*-test, *p* < 0.05). Moreover; two findings are worth consideration: (1) Despite the high variability between subjects in mean correlation values between TPN and PCC for uncorrected fcMRI maps, sGSR processing resulted in anticorrelations that did not present a significant variability in magnitude across subjects. (2) After dGSR, the anticorrelations were still present but their magnitudes were attenuated and a variability among subjects could easily be observed. Results of a group level fixed effects analysis after dGSR and sGSR methods for negative and positive correlations with the PCC are shown in Figure 11 (corrected for multiple comparisons at a significance level of *p* < 0.05). When compared to functional connectivity measures obtained with dGSR at the group level, sGSR increases the spatial extent and magnitude of significant negative correlations in TPN regions, while it reduces the spatial extent and magnitude of positive correlations in DMN regions.

## Discussion

The presented study provides evidence that correcting resting state BOLD-fMRI signals for blood arrival time enhances functional connectivity analysis. The low frequency systemic component of resting state fMRI-BOLD signal moves with blood flow within the cerebral circulation mechanism. The dynamic propagation of these systemic LFOs through the cerebral vasculature can be modeled by applying voxel-specific optimal time delays to the global signal. The close resemblance of the time delay map obtained in this study to the cerebral blood circulation maps obtained in previous studies indicate that, voxel-specific optimal alignment of the global signal regressor presents a physiologically appropriate approach for modeling the moving systemic LFO component of resting state BOLD signals. We present physiological and quantitative evidence that modeling and removing systemic noise in resting state fMRI data (0.01–0.1 Hz) by accounting for the relative arrival time of sLFOs in each voxel will be a more plausible approach for optimal denoising when compared to the conventional sGSR method. In the following paragraphs, we discuss the performance of dGSR and sGSR in terms of reducing systemic variance, and enhancing the specificity and sensitivity of positive and negative correlations with PCC, a hub region in the DMN. In addition, we discuss some of the potential mechanisms by which the performance of sGSR and dGSR may differ.

### Explained variance

Our results suggest that the nonhomogeneous distribution of global systemic effects is captured to a greater extent when these systemic effects are modeled and removed with the dGSR method. We report significant improvements in systemic noise removal as reflected in the % explained variance parameter when compared to sGSR. Removing the global signal by accounting for its optimal time delay with respect to local signals explains additional variance in a majority of brain voxels, with up to 20% additional explained variance in highly perfused regions and near large blood vessels (Figure 5B). Correcting for the relative arrival time of the blood borne physiological signal in each voxel enhances the amount of BOLD signal variance being modeled and removed when compared to conventional static global regression (Figure 5A).

Applying voxel-specific optimal time delays to the global signal results in spatial maps that reflect the dynamic propagation of sLFOs with bulk blood, and that show a good correspondence in both cerebral blood flow patterns and circulation time (Figure 4) to the delay maps obtained in previous work through the use of auxiliary peripheral NIRS recordings (Tong and Frederick, 2012; Tong et al., 2013) and fMRI based recursive regressors (Tong and Frederick, 2014) and which have recently been validated to be in compliance with gold-standard DSC cerebral blood flow measurements (Tong et al., 2016). In contrast, applying dynamic regression procedure with subject non-specific low frequency regressors (sham global or sinusoid) can neither explain the variance in the data nor generate these circulatory maps. These observations highlight the fact that global signal from rsfMRI contains a considerable amount of sLFOs intrinsic to subject physiology and optimally delaying this proxy systemic regressor is a physiologically justified method of modeling the propagating sLFO component of resting state BOLD signals.

### Sensitivity and specificity of negative and positive correlations

Our results demonstrated that a preprocessing pipeline utilizing dGSR after bandpass filtering and removal of motion parameters improves both sensitivity and specificity for detecting TPN and DMN regions in seed based fcMRI analysis when compared to the standard sGSR approach. When no physiological noise correction is applied, false positive correlations between PCC and reference regions exist due to effects of common systemic noise (Figure 7C). These positive correlation measures are spurious or at least inflated since reference regions are chosen based on no expectation of significant correlation with the PCC. However, when sGSR noise reduction is applied to correct for systemic noise effects, false negative correlations may result between PCC and reference areas in visual cortex as we observed at the group level. Significant negative correlations between PCC and reference regions after sGSR indicate that sGSR most probably inflated negative correlation measures and may lead to spurious negative correlations in regions where no significant correlation is expected. Instead, applying a voxel-specific optimal time delay to global signal prior to GSR from voxel-wise data (dGSR) attenuated artificial negative connectivity measures while still removing physiological noise effectively (no association is found between PCC and reference areas).

For positively correlated regions with PCC ROI (DMN or TNN regions), examining sensitivity at the group (Figure 7A) and single subject level (Figure 9A) together with the specificity metric (Figure 8A) suggests that, positive connectivity measures are underestimated when sGSR is applied instead of dGSR. This result is in accordance with the rationale of Murphy et al. (2009) who discussed extensively the mathematical bias of sGSR method for shifting the distribution of correlation coefficients toward more negative values. As predicted by Murphy et al. (2009), sGSR yielded stronger anticorrelations with PCC in TPN region ROIs both at the group (Figure 7B) and single subject level (Figure 9B). However; the specificities of these anticorrelations were quite low (Figure 8A) because of the inflated negative correlations with the reference regions (Figure 7C, note how inflated negative measures of reference regions necessarily decrease the specificity metric in Equation 1). After dGSR, the anticorrelations are still present but with reduced magnitude and with considerably higher specificity (Figure 7B).

Previous work by Fox et al. (2009) and Weissenbacher et al. (2009) reported increases in the specificity of fcMRI maps of several functional systems after employing sGSR. However, it should be noted that they found specificity improvements only for systems that would exhibit positive correlations with the selected seed ROIs. Chai et al. (2012) compared the specificity of sGSR to a component base noise reduction method (CompCor; Behzadi et al., 2007) for detecting positive and negative correlations with a DMN seed region. They reported higher specificity for CompCor method for positive correlations; however the specificity of both methods were found to be similar and lower for negative correlations than for positive correlations. In our study, specificity of dGSR for detecting both positive and negative correlations was considerably higher than the sGSR method.

Anticorrelations between the DMN and TPN regions are still present when the relative arrival time of sLFOs are taken into account with dGSR method, but to a lesser extent in magnitude. Our results suggest that anticorrelation between DMN and TPN does have some neural origin; however, accounting for voxel-specific time delays in global systemic effects with the dGSR method can attenuate artificial false negative correlations introduced by sGSR while providing relatively higher specificity for detecting functional network strengths.

### Inferences on sensitivity and specificity from time delay simulations

To demonstrate how sGSR can introduce spurious positive and negative correlation measures in seed based fcMRI analyses, we adopted a time delay simulation scheme very similar to the phase simulations presented by Murphy et al. (2009). These simulations demonstrated the improved efficacy of the dGSR approach in (i) preventing inflated negative and positive correlation measures (reference voxels outside the depicted network have no significant correlations with the seed ROI after dGSR), (ii) eliminating artifactual anticorrelation measures as well as (iii) preserving true positive correlation measures between functionally related regions. These simulations also demonstrate that the relative arrival time of systemic fluctuations in a voxel can bias those regions to become anticorrelated or uncorrelated unless arrival time is modeled.

In a very simplistic manner, we propose the resting state BOLD signal Y_n_(t) from a voxel *n* can be modeled as a linear sum of a neuronal LFO component N(t), a dynamic systemic LFO component S(t) which arrives in each voxel with a time delay ΔT and some undefined process noise ν_n_ as:

(2) $$\begin{array}{l} {\text{Y}}_{\text{n}}\left(\text{t}\right)=\text{N}\left(\text{t}\right)+\text{S}\left(\text{t}+\Delta {\text{T}}_{\text{n}}\right)+\nu_{\text{n},} \end{array}$$

If a systemic noise modeling regressor is optimally aligned with Y(t) prior to regression, it may better model the relative arrival time of the systemic LFO component in each voxel time series. Indeed, subtracting a systemic regressor with an incorrect time shift with sGSR methods likely results in a distortion of the denoised signal from the neural activity related component N(t) in a majority of voxels, leading to suboptimal performance. Both the reduction in the magnitude of spurious correlations in real and simulated data, and the increase in the specificity of neuronal activity related functional connectivity measures with the dGSR method emphasize the importance of including temporal delay information for sLFOs in fcMRI noise removal procedures.

The close resemblance of time delay maps obtained with dynamic global regressors to the cerebral blood circulation maps obtained in previous studies indicate that optimally delayed global regressors are a reasonable model of the propagating systemic LFO component of BOLD signals. It is well known that systemic noise accounts for up to 30% of the signal variance in fMRI data (Frederick et al., 2012). We believe the introduction of spurious negative or positive correlation measures with sGSR resulted from regressing out a nuisance regressor which is improperly aligned with the systemic component in each voxel time series.

### The spatial extent of negative and positive correlations

We examined the spatial extent of negative and positive correlations with PCC when dGSR is applied in lieu of the conventional sGSR for a better interpretation of the artifactually inflated anticorrelations and spurious negative correlation measures introduced. sGSR results in increased spatial extent and magnitude of negative correlations while reducing those of positive correlations with PCC when compared to dGSR. When dGSR is used, negatively correlated regions overlap with those found after sGSR, however they are more localized and their magnitude is diminished (Figure 9). In accordance with similar findings by Chang et al. (2009) and Murphy et al. (2009), these observations confirm that sGSR forces the distribution of correlations toward negative measures, necessarily inflating the magnitude and extent of negative connectivity measures, hence introducing spurious anticorrelations. This is not the case for dGSR method.

In summary, performing sGSR for physiological noise removal from fcFMRI data necessarily results in some artifactual anticorrelations, as seen most clearly in PCC correlations with reference regions. In contrast, dGSR may correctly remove physiological noise without introducing these effects, as confirmed by reference regions uncorrelated with PCC. dGSR preserves positive correlations with PCC ROI time series in task negative regions while attenuating some of the inflated artificial negative correlations in task positive regions. Anticorrelations between the DMN and TPN regions remain present when the relative arrival time of sLFOs are taken into account, but to a lesser extent. We conclude that anticorrelations in resting state fcMRI maps cannot be fully attributed to sGSR methodology and may be neuronal in origin.

### The origin and physiology of the global signal

An important concern about dGSR or any other denoising approach utilizing global signal is the possibility that global signal contains neuronal information. In a study by Scholvinck et al. (2010), microelectrode recordings in anesthetized monkeys from single cortical sites at rest were shown to display spatially widespread cross-correlations with the fMRI BOLD signals over large regions of the cerebral cortex. Although the global signal is dominated by systemic oscillations (Birn et al., 2006; Lund et al., 2006; Beall and Lowe, 2007), there is a high chance that it also includes contributions from neurophysiological activity. The neuronally derived component of the global signal could reflect a specific spontaneous fluctuation which may arise from a shared covariation in firing rate of neurons, as discussed in Scholvinck et al. (2010), or it could simply be an average of neuronal activity throughout the brain (Murphy et al., 2009). Nevertheless; regardless of its origin, the neuronal component of global signal still poses an undesired variance since it is common to all functional networks (Fox et al., 2009; Carbonell et al., 2011; Power et al., 2014).

Conventional GSR (sGSR) has been shown to facilitate removal of these undesired shared components among functional networks which obscure network specific resting-state fluctuations and has been reported to enhance the detection of network specific fluctuations (Nir et al., 2006; Fox et al., 2009; Carbonell et al., 2011; Power et al., 2014; Fang et al., 2016) as well as interactions known to exist at the neurophysiological level in animal models (Dawson et al., 2013). In addition, in a recent study performed by Keller et al. (2013) on human subjects; applying sGSR increased the correspondence between resting state fMRI BOLD signals and electrophysiological high gamma power (HGP) signals obtained from direct cortical surface measurements, suggesting increased correspondence between neuronal and hemodynamic measures after sGSR. In their study, Keller et al. (2013) reported that sGSR decreased the variability in discriminability between subjects while increasing the sensitivity and specificity of BOLD intrinsic functional connectivity with regard to both correlated and anticorrelated HGP intrinsic functional connectivity. Moreover; performing sGSR before computing HGP intrinsic connectivity measures increased their correspondence with functional connectivity measures which may potentially reflect the benefits of removing an obscuring global signal from both the neuronal and BOLD data.

The rationale behind GSR approaches comes from the observation that global signal from resting state fMRI data is dominated by systemic physiological effects of non-neuronal origin (Biswal et al., 1996; Kruger and Glover, 2001; Kruger et al., 2001; Wise et al., 2004; Triantafyllou et al., 2005; Birn et al., 2006; Lund et al., 2006; Beall and Lowe, 2007; Bianciardi et al., 2009; Murphy et al., 2009). Although some fraction of the global signal might be neuronally derived (Scholvinck et al., 2010); it is certain that a large fraction is attributed to systemic physiological effects originating from fluctuations in the partial pressure of end-tidal CO_2_ (Wise et al., 2004; Birn et al., 2006) and oscillations in vascular tone related to pulsatile changes in arterial blood pressure and respiration (Kruger and Glover, 2001; Kruger et al., 2001; Birn et al., 2006), which are independent of changes in local neuronal activity (Murphy et al., 2013). In addition; the spatial characteristics of blood arrival times derived with voxel-specific optimal alignment of the global signal were shown to be very similar to the cerebral blood flow patterns obtained with DSC in previous multimodal studies performed by other groups (Amemiya et al., 2014; Christen et al., 2015) which corroborate the idea that the major component of global signal is attributed to cerebrovascular circulatory effects. The time delays observed among different brain regions are between 6 and 8 s, implicating a circulatory rather than neuronal origin.

When interpreting the correlation between global signal from fMRI and the fingertip HbO signal from NIRS measurements (or any other physiological noise modeling regressor obtained from the brain or peripheral tissue); one should keep in mind the possibility that autonomic nervous system (ANS) activity may shape the neural responses of certain brain regions as well the fingertip HbO signal, leading to the possibility that the high correlations observed between two signals may also be partially attributed to not only non-neuronal effects but also to shared neuronal contributions. Indeed, a recent study combining skin conductance measurements from the peripheral sites and resting state BOLD fMRI measurements showed that spontaneous BOLD signal fluctuations in key nodes of resting state networks are associated with changes in skin conductance response (SCR) measured from the toe (Fan et al., 2012). However; we should note that there are subtle differences between the underlying neurobiological mechanisms forming the SCR and fingertip HbO signals. Changes in SCR are directly and solely related to sympathetic activity of sweat glands and can be regarded as accurate correlates of afferent neural activity of the ANS. Meanwhile; ANS also regulates cardiac output, blood flow, and blood pressure with feedback and feed-forward regulatory mechanisms, which will in turn, result in variations in the fingertip HbO signal as well as brain BOLD signals (Marmarelis et al., 2013). Variations in the fingertip HbO signal are shaped by multiple putative contributors from systemic physiology such as vessel diameter changes or vasomotion (Aalkjaer et al., 2011; Sassaroli et al., 2012), systemic changes in arterial blood pressure known as Mayer waves and variations in respiratory volume and cardiac rate (Glover et al., 2000; Birn et al., 2008a,b). Therefore, the underlying neurobiological mechanisms forming the SCR and fingertip HbO signals differ in the sense that while SCR is governed directly by afferent activity of neurons, the fingertip HbO signal is an indirect measure of the ANS modulating systemic circulation signals from the heart. Similar to the systemic physiological contributors to fingertip HbO signal, spontaneous low frequency BOLD fluctuations in the brain are influenced by upstream vascular changes related to pulsatile blood pressure and respiratory oscillations (Huijbers et al., 2014) which are independent of changes in local neuronal activity (Murphy et al., 2013). Hence; while the global signal might have neuronal contributions, it could at the same time be governed by non-neuronal vascular effects produced from upstream changes in cerebral hemodynamics.

Supporting this notion of upstream vascular effects on BOLD signals at low frequencies, recent work using high temporal resolution transcranial Doppler flowmetry (TCD) and NIRS in human subjects showed that spontaneous fluctuations in cerebral microcirculation are influenced to a great extent by systemic circulation and upstream changes in cerebral blood flow (Zhang et al., 1998; Tarumi et al., 2014; Vermeij et al., 2014). Specifically, it has been shown that both systemic arterial pressure and cerebral blood flow volume measured in the basal cerebral arteries have similar power spectral distributions with predominant fluctuations at frequencies < 0.1 Hz (Zhang et al., 1998, 2000). Furthermore, perturbations in low-frequency cerebral blood flow velocity in the middle cerebral artery due to posture changes could be observed in cerebral microcirculation (Tarumi et al., 2014) and mathematical modeling indicated that the relationship between dynamic changes in arterial pressure and cerebral blood flow is modulated by arterial CO_2_ through complicated feed-back and feed-forward regulatory mechanisms (Marmarelis et al., 2013). These physiological studies suggest that spontaneous low-frequency BOLD fluctuations at < 0.1 Hz are likely to be induced at least partially by changes in upstream cerebral hemodynamics independent of regional neuronal activity which will in turn affect whole brain vascular hemodynamics and dominate the global signal.

One possibility for dissociating physiological noise dominated by upstream hemodynamics from neural signals arises when these components can be spatially and/or temporally separated. In previous work, by applying a range of time delays to the reference fingertip NIRS LFO signal, it was observed that the systemic LFOs modeled through peripheral NIRS HbO regressors evolved temporally through the brain in a manner that suggests a circulatory rather than neuronal origin, and a global rather than local character. The temporal evolution of the systemic LFOs modeled through, peripheral NIRS regressors (Tong et al., 2012), recursive regressors from the brain (Tong and Frederick, 2014) and global signal regressor used in this study and in recent studies by other groups (Lv et al., 2013; Amemiya et al., 2014; Christen et al., 2015) have time lags in the range of 6–8 s which would be too long for neuronally induced time lags, and the spatial pattern of these time lags appears to closely follow the cerebral circulatory system from arteries to the gray matter (where most blood vessels reside), to the venous system and finally in white matter. The spatial patterns of time delays obtained by optimally aligning the global signal showed a good consistency with the time delays of cerebrovascular perfusion that were measured using DSC enhanced perfusion imaging (Lv et al., 2013; Amemiya et al., 2014; Christen et al., 2015). In all of these studies, the component of the LFOs that moved with the blood was already present before the blood reached gray matter. The spatio-temporal characteristics of time delays observed in this study and in previous studies suggest a major source of circulatory origin for the sLFO component of BOLD fluctuations modeled with optimally aligned global signal.

To summarize; in accordance with previous studies, our findings suggest a source for moving component of LFOs (i.e., sLFOs) outside of the brain and imply that a major component of the LFOs detected by both peripheral NIRS and brain-fMRI measurements arises from the propagation of endogenous global blood flow and oxygenation fluctuations through the cerebral vasculature, rather than from local variations in neuronal activation or localized cerebral blood flow changes. However, these findings do not necessarily preclude the contribution of neural activity in global signal. There is always a possibility of a neuronal contribution to the global signal, but in this study we assumed global signal is dominated by physiological noise of non-neural origin as commonly proposed in a variety of studies. This assumption is most likely valid as averaging signals from all voxels across the brain will enhance common signals while canceling out localized effects.

### Limitations of the study and recommendations for future work

There is a growing concern that GSR could introduce confounds into between-group connectivity comparisons (Saad et al., 2012; Yang et al., 2014), especially in cases where one of the clinical groups exhibits greater resting state signal variability (Yang et al., 2014). In a recent simulation study by Saad et al. (2012), it has been pointed out that GSR can artificially induce group differences if the global signal is unequally distributed in comparison groups (Saad et al., 2012; Yang et al., 2014) found that the variability of the resting-state global brain signal was greater in patients with schizophrenia as compared to matched controls. We agree that global signal should not be automatically removed in some clinical studies where the global signal variance is significantly different between groups especially when comparing schizophrenia patients with control or other disease/clinical populations (Hahamy et al., 2014; Yang et al., 2014). However; it is important to note that the correlation patterns after GSR were not necessarily distorted in similar studies that compared neuropsychiatric populations including schizophrenia with other groups. Anticevic et al. (2015) examined prefrontal cortex resting state functional differences between patients diagnosed with early course schizophrenia and healthy matched controls both with and without GSR implemented. They reported that all clinical effects remained unchanged and found no alteration in any of their reported patterns. Similarly, several other groups investigated the effect of applying and not applying GSR on their resting state fMRI data collected from healthy controls and clinical populations to investigate the possible impact of this step on reported clinical effects. They concluded that their results did not depend on the effect of removing or not the global signal from the time series data (Fang et al., 2013; Kim et al., 2014; Liu et al., 2014; Alonso Montes et al., 2015; Grewen et al., 2015; Liang et al., 2015; Nair et al., 2015). Group-differences and trends were highly consistent with those reported using data treated with GSR. While GSR may in principle pose a risk of distorting fcMRI group comparisons (Saad et al., 2012); due to the continuing debate on the virtues of GSR (Power et al., 2014), performing clinical analyses both with and without GSR has been suggested by many researchers to ensure robustness of main effects in such cases. We suggest that dGSR method should be used with caution in clinical connectivity analyses especially when the variability between global signals in different groups are relatively high or the inherent interaction structure of the brain is known to be altered in two different states that are used for comparison.

Currently, there is not a clear consensus with regard to the best approach for addressing global systemic artifacts in fMRI data. Many studies still continue to use sGSR, while an increasing number of studies have begun to employ alternative methods such as CompCor (Behzadi et al., 2007), PESTICA (Beall and Lowe, 2007), CORSICA (Perlbarg et al., 2007), PSTCor (Anderson et al., 2011), and FIX (Griffanti et al., 2014), which estimate physiological noise contribution from the data itself. Among these methods, CompCor method was proposed to correct for physiological noise by regressing out principle components from noise regions of interest (ROIs) in white matter (WM) and cerebrospinal fluid (CSF) regions (Behzadi et al., 2007). We should note that CompCor is designed for removing high frequency noise such as cardiac and respiratory effects and is biased toward higher frequencies. Our method is preferentially sensitive to noise in the low frequency range, the band of interest for studying connectivity. We believe combining CompCor with dGSR in future studies will be a valuable approach for addressing confounds in all frequency ranges for data acquisition protocols where the TR is not short enough to remove aliased cardiac and respiratory signals in the low frequency range.

We propose dGSR as a reasonable alternative to sGSR, and show its efficacy in avoiding sGSR related inflated and/or false negative correlations of spurious origin, that have been demonstrated both here and in previous studies. In this preliminary analysis, we observed that anticorrelations between the DMN and TPN are present also with dGSR but with reduced magnitude and higher specificity. Therefore, our results suggest that these anticorrelations may reflect underlying biology rather than being simply a mathematical artifact. For future work, we plan to apply this novel dynamic regression strategy to analyze large scale resting state fMRI data sets collected from healthy controls and different patient populations to exploit the potential of this method more thoroughly and investigate the interactions between task positive and TNNs.

Effective physiological denoising is an important step in fcMRI analysis. Our previous work indicated that sLFOs are major contributors to the resting state BOLD signal and must be considered to be an independent physiological noise source instead of an aliasing artifact. sLFOs measured by NIRS at the periphery, or derived recursively from the resting state BOLD-fMRI measurements, represent the same propagating biological signal (Tong et al., 2012; Tong and Frederick, 2014). These sLFOs carry distinct temporal and spatial information (Hocke et al., 2016) which is different from LFOs derived with models using the respiratory and cardiac signals (Birn et al., 2008b; Chang et al., 2009). In this study, we demonstrate the utility of a voxel-specific temporal alignment for the global signal from fMRI prior to regression, taking into consideration the dynamic propagation of global systemic effects throughout the brain. We plan to explore the extent of correspondance between peripheral low frequency NIRS oxygenation measurements and resting state global fMRI signal in a larger scale data set and compare their noise removal performance.

The dGSR method takes into account a fundamental property of vascular dynamics that clearly defines the nature of physiological noise in resting state fMRI data. This fundamental characteristic has been validated to be reflected in temporal delays of the global signal both in this work and in other studies (Lv et al., 2013; Amemiya et al., 2014; Christen et al., 2015; Tong et al., 2016). Our results indicate that physiological noise removal methods for fcMRI analysis should incorporate temporal information about the dynamic propagation of systemic noise sources. Many physiological fluctuations such as sLFOs, respiration, and cardiac pulsation, travel with different speeds inside the blood vessels throughout the body. Since these signals are dynamic, they cannot be effectively removed from voxel-wise BOLD-fMRI signals using a static regressor. The RIPTiDe method can characterize the voxel-specific temporal delay of any input physiological regressor throughout the brain (Frederick et al., 2012). We continue to refine this noise removal method for both resting state and task fMRI studies. While dGSR has the simple advantage of being parsimonious, we further aim to compare its performance to other physiological noise removal methods in future work.

## Conclusion

The current study demonstrates that the global signal can be used as a proxy systemic regressor to model the dynamic passage of systemic low frequency fluctuations through the cerebral vasculature. Our findings suggest that a preprocessing pipeline using dGSR after band-pass filtering and regressing out realignment parameters provides better sensitivity in terms of functional connectivity strength and enhanced specificity while reducing spurious noise more effectively. We conclude that incorporating time delay information for sLFOs into global noise removal strategies is crucially important for optimal noise removal from resting state fcMRI maps. The dGSR approach matches the underlying dynamic systemic physiology to a good extent, relies only on the BOLD data itself, and does not require any additional physiological measurement, so it can be applied retrospectively to any resting state data set to improve the modeling and removal of physiological variance.

## Author contributions

SE prepared the original manuscript and figures, performed statistical analysis and interpreted the results. BF designed and conducted experiments. YT and BF participated in the design of the time delay processing methodology and interpreted the results. LH, KL, and BF participated in data acquisition and quality control. All participated in manuscript review and interpretation of the study in the context of literature.

### Conflict of interest statement

The authors declare that the research was conducted in the absence of any commercial or financial relationships that could be construed as a potential conflict of interest.

## Supplementary material

The time delay analysis software used in this work can be downloaded from https://github.com/bbfrederick/rapidtide.
