# Supplementary material for: Correcting for Blood Arrival Time in Global Mean Regression Enhances Functional Connectivity Analysis of Resting State fMRI-BOLD Signals
Source: Front Hum Neurosci. 2016 Jun 28;10:311. doi: 10.3389/fnhum.2016.00311 (PMC4923135; doi:10.3389/fnhum.2016.00311)
Supplement: Supplementary file 1 [file Image1.PDF]

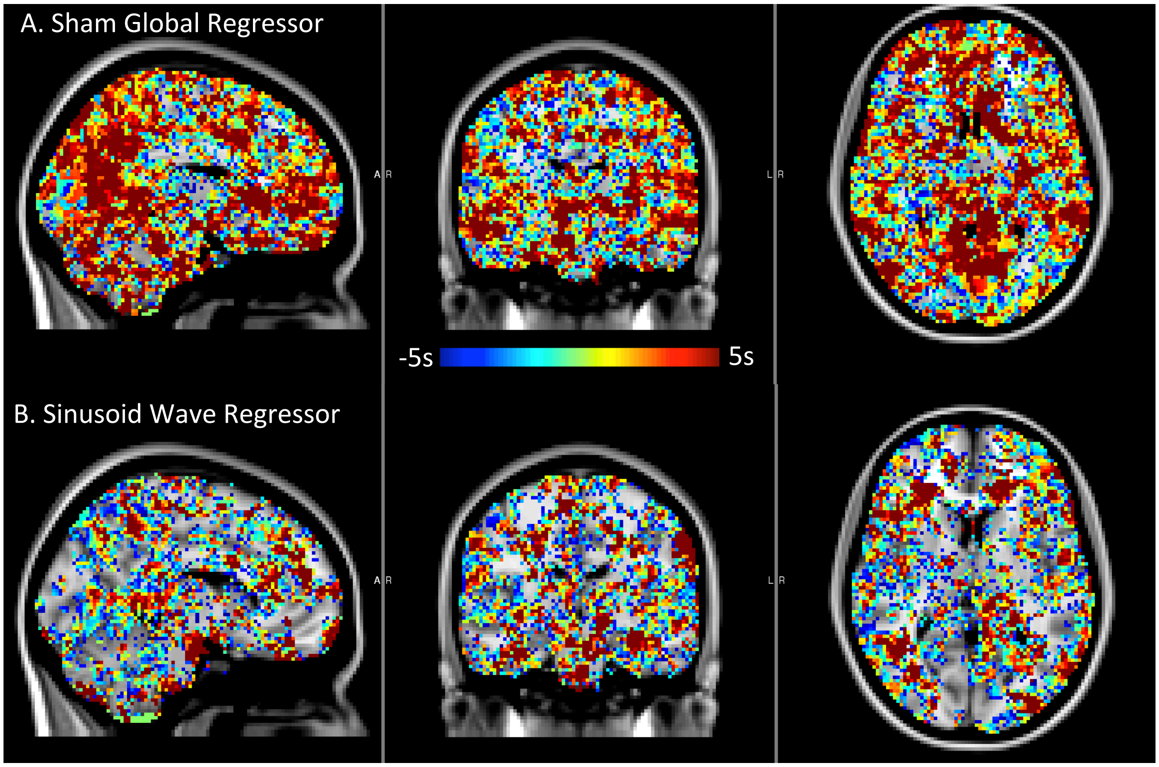

Figure S.1 Group average of time delay maps obtained by optimally delaying a) sham global regressors (using another subject's global regressor) and b) a sinusoid wave regressor (f=0.1 Hz). One question with the dGSR approach can be whether the same systemic variance removal performance and similar circulatory delay patterns can be obtained by optimally delaying any randomly selected aperiodic or periodic low frequency signal for each voxel time series. Applying dynamic regression procedure with subject non-specific low frequency regressors (sham global or sinusoid) can neither explain the systemic variance in the data to a good extent (Fig.6) nor generate time delay maps that resemble cerebral circulation patterns. Most of the cross-correlations obtained between voxel time series and sham global regressors or periodic sinusoid wave regressors did not pass the statistical significance threshold. For the surviving voxels which had significant correlation with the optimally aligned sham global or sine wave regressors, the mean time delay maps did not produce any spatial coherence. This highlights the fact that global signal from resting state fMRI contains a considerable amount of sLFOs intrinsic to subject physiology and optimally delaying this proxy systemic regressor is a physiologically relevant approach for modeling the propagation of sLFO component of resting state BOLD signals with cerebral blood circulation.
