# Supplementary material for: Correcting for Blood Arrival Time in Global Mean Regression Enhances Functional Connectivity Analysis of Resting State fMRI-BOLD Signals
Source: Front Hum Neurosci. 2016 Jun 28;10:311. doi: 10.3389/fnhum.2016.00311 (PMC4923135; doi:10.3389/fnhum.2016.00311)
Supplement: Supplementary file 2 [file Image2.PDF]

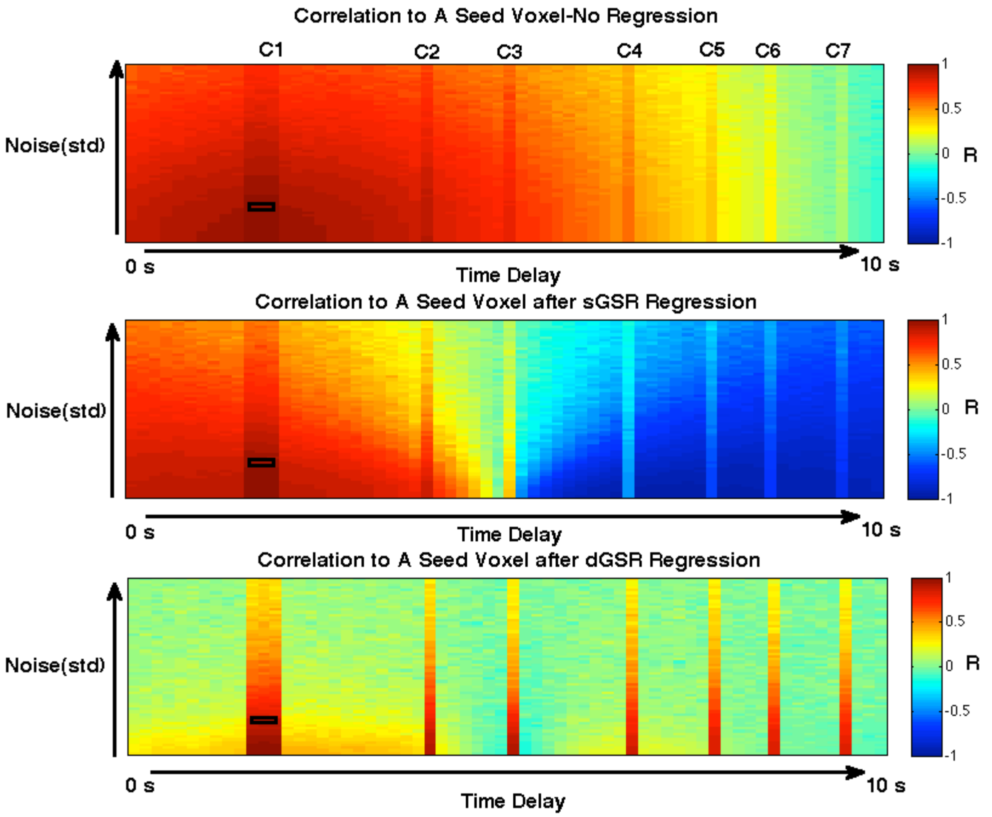

Figure S.2 The results of the phase simulation are shown. Time delay simulations were repeated by keeping all procedures the same; however choosing a single frequency periodic sinusoidal wave (0.1 Hz) range whose phase varied progressively in the x-axis from 0 to $\pi/4$ (as in the original work of Murphy et al., 2009) to represent the systemic component of resting state low frequency oscillations. We observed the improvement in extracting the true correlations and preventing false negative and positive correlation measures with the dGSR method in a consistent manner with the aperiodic low frequency systemic signal example presented in Figure 9.

This figure demonstrates that small phase differences between systemic components of different voxel time series may lead to false negative correlations with seed ROI when sGSR is performed. The sGSR method is 1) suboptimal for removing false positive correlations from some reference regions, 2) underestimates true positive neuronal correlations in some regions (C2 and C3) and 3) may introduce false anticorrelations in some regions.

When the dGSR method is applied; 1) the reference voxels which are expected to have no significant negative or positive correlations with the seed ROI (voxels outside the depicted network in regions C1-C7 which have no coherent neuronal content) were

uncorrelated after regression. This result is indicative of the ability of dGSR to attenuate both false positive and false negative correlation measures. 2) The true correlations with voxels which contained coherent neuronal oscillations (C1-C7 regions) were preserved to a good extent as well. These findings demonstrate evidence for the improved efficacy of the dGSR approach in i) preventing inflated spurious negative and positive correlation measures, ii) preserving true positive correlations, and ii) eliminating artifactual anticorrelations.
